# Genome-Wide Analysis of Secondary Metabolite Gene Clusters in O*phiostoma ulmi* and *Ophiostoma novo-ulmi* Reveals a Fujikurin-Like Gene Cluster with a Putative Role in Infection

**DOI:** 10.3389/fmicb.2017.01063

**Published:** 2017-06-13

**Authors:** Nicolau Sbaraini, Fábio C. Andreis, Claudia E. Thompson, Rafael L. M. Guedes, Ângela Junges, Thais Campos, Charley C. Staats, Marilene H. Vainstein, Ana T. Ribeiro de Vasconcelos, Augusto Schrank

**Affiliations:** ^1^Rede Avançada em Biologia ComputacionalPetrópolis, Brazil; ^2^Centro de Biotecnologia, Programa de Pós-Graduação em Biologia Celular e Molecular, Universidade Federal do Rio Grande do SulPorto Alegre, Brazil; ^3^Laboratório Nacional de Computação CientíficaPetrópolis, Brazil

**Keywords:** *Ophiostoma ulmi*, *Ophiostoma novo-ulmi*, Ophiostomataceae, secondary metabolite biosynthetic gene clusters, infection process, Dutch elm disease

## Abstract

The emergence of new microbial pathogens can result in destructive outbreaks, since their hosts have limited resistance and pathogens may be excessively aggressive. Described as the major ecological incident of the twentieth century, Dutch elm disease, caused by ascomycete fungi from the *Ophiostoma* genus, has caused a significant decline in elm tree populations (*Ulmus* sp.) in North America and Europe. Genome sequencing of the two main causative agents of Dutch elm disease (*Ophiostoma ulmi* and *Ophiostoma novo-ulmi*), along with closely related species with different lifestyles, allows for unique comparisons to be made to identify how pathogens and virulence determinants have emerged. Among several established virulence determinants, secondary metabolites (SMs) have been suggested to play significant roles during phytopathogen infection. Interestingly, the secondary metabolism of Dutch elm pathogens remains almost unexplored, and little is known about how SM biosynthetic genes are organized in these species. To better understand the metabolic potential of *O. ulmi* and *O. novo-ulmi*, we performed a deep survey and description of SM biosynthetic gene clusters (BGCs) in these species and assessed their conservation among eight species from the Ophiostomataceae family. Among 19 identified BGCs, a fujikurin-like gene cluster (OpPKS8) was unique to Dutch elm pathogens. Phylogenetic analysis revealed that orthologs for this gene cluster are widespread among phytopathogens and plant-associated fungi, suggesting that OpPKS8 may have been horizontally acquired by the *Ophiostoma* genus. Moreover, the detailed identification of several BGCs paves the way for future in-depth research and supports the potential impact of secondary metabolism on *Ophiostoma* genus’ lifestyle.

## Introduction

*Ophiostoma ulmi* and *Ophiostoma novo-ulmi* (Ophiostomatales: Ophiostomataceae) are phytopathogenic fungi responsible for Dutch elm disease (DED), which was responsible for several catastrophic epidemics affecting elm trees (*Ulmus* sp.) during the 20th century in Europe and North America (Brasier, 2001). Interestingly, DED was unknown in these continents before 1900, and DED pathogens are believed to be invasive species introduced by human activity (Brasier, 2001). DED spreads within the tree’s vascular system and is transmitted from diseased to healthy elms by elm bark beetles from the *Scolytus* and *Hylurgopinus* genera (Brasier, 2001; Naruzawa and Bernier, 2014). A prominent characteristic of DED pathogens is yeast-mycelium dimorphism, which is predicted to be essential for successful infection and dispersion to new, healthy trees (Nigg et al., 2015; Wedge et al., 2016). Unlike these well-known cellular aspects of fungal infection, molecular mechanisms such as virulence determinant genes are almost unexplored in DED pathogens. In order to provide a framework to explore this matter, the genomes of *O. ulmi* and *O. novo-ulmi* have recently been published (Khoshraftar et al., 2013; Comeau et al., 2015). Additionally, the genomes of a closely related saprophytic species (*Ophiostoma piceae*) and species of the same family with different pathogenic/lifestyle traits (*Sporothrix schenckii*, *Sporothrix brasiliensis*, *Sporothrix pallida*, *Grosmannia clavigera*, *Leptographium lundbergii*, *Leptographium procerum*, and *Graphilbum fragrans*) are also available (DiGuistini et al., 2011; Haridas et al., 2013; Teixeira et al., 2014; van der Nest et al., 2014; Wingfield et al., 2015a,b, 2016; D’Alessandro et al., 2016). This information allows for unique comparisons to be made between species to highlight putative virulence determinants in DED pathogens.

Among the fungal virulence determinants already described (e.g., hydrolytic enzymes, specialized infection structures, and shedding of decoy components), secondary metabolites (SMs) are known to have important functions during the infection process for several pathogenic fungi due to their diverse biological activities (Osbourn, 2010; Gibson et al., 2014; Keller, 2015). For example, the causal agent of gray leaf spot, *Alternaria brassicae*, and that of southern corn leaf blight, *Cochliobolus heterostrophus*, produce destruxin B and T-toxin that induce leaf chlorosis (Parada et al., 2007) and tissue necrosis (Yang et al., 1996; Inderbitzin et al., 2010), respectively.

In fungi, genes required for SM biosynthesis are usually clustered. The spatial proximity of SM production-related genes in biosynthetic gene clusters (BGCs) minimizes the number of regulatory steps in the biosynthetic machinery, thereby contributing to physiological optimization (Gacek and Strauss, 2012). These BGCs usually contain backbone genes such as polyketide synthases (PKS) and non-ribosomal peptide synthetases (NRPS), as well as adjacent genes that assist in regulation, transport, and metabolite trimming (Inglis et al., 2013; Lazarus et al., 2014). The increasing number of fungal genome sequences, combined with BGC prediction tools, has revealed that filamentous fungi can produce far greater numbers of low molecular weight compounds than previously estimated, and such diversity of silent metabolites (which are not accessible under normal laboratory culture conditions) reflects habitat complexity and represents significant scientific and commercial opportunities (Brakhage, 2013; Yaegashi et al., 2014; Keller, 2015; Rutledge and Challis, 2015).

Furthermore, it has been proposed that clustering increases the survival of SM genes, since BGCs partially depend on horizontal gene transfer (HGT) for their dispersal (Walton, 2000). Consistent with this hypothesis, several horizontally transferred BGCs have been described in recent years (Khaldi et al., 2008; Slot and Rokas, 2011). HGT events for BGCs have also been linked to the success of emergent pathogens. The poplar tree pathogen *Mycosphaerella populorum*, for instance, putatively acquired, from an unknown donor, a chaetoglobosin-like gene cluster that is potentially involved in poplar infection (Dhillon et al., 2015). Notably, the mechanisms that lead to foreign DNA uptake and HGT events between non-related fungal species are not fully understood (Fitzpatrick, 2012).

Dutch elm disease species are promising models to study the impact of invasive species and the evolution and adaptation to new niches, in view of the success and persistence of these pathogens in the environment. Furthermore, it is reasonable to propose that SMs may assist in the infection process of DED pathogens. However, secondary metabolism and genes enrolled in SM production are virtually unexplored in the *Ophiostoma* genus and Ophiostomataceae family. To investigate DED-related BGCs, we explored the genomes of the *O. ulmi* strain W9 and the *O. novo-ulmi* strain H327. Subsequently, we assessed the conservation of BGCs and related genes identified within the eight available Ophiostomataceae family genomes. Interestingly, a specific PKS BGC (OpPKS8) was conserved only in DED pathogens and was absent in other members of the Ophiostomataceae family. Through phylogenetic inference and comparative genomic analyses, we showed that this cluster may have been horizontally acquired by DED pathogens. Additionally, orthologs for this BGC were found in several plant-associated fungi, which supports a putative role for the products of this gene cluster in phytopathogenic infection.

## Materials and Methods

### Genomes and Predictions of Secondary Metabolite Biosynthetic Gene Clusters

All fungal genomes were downloaded from the NCBI Genome Database, with the exception of the *O. ulmi* W9 genome (downloaded from http://www.moseslab.csb.utoronto.ca/o.ulmi/). The descriptions and accession numbers are displayed in Supplementary Table 1 The predicted proteins/protein annotation for *O. novo-ulmi* H327 is available as supporting information from Comeau et al. (2015), and their proposed nomenclature was used throughout the text. To assess the completeness and quality of genome sequence assembly/predicted proteins, BUSCO version 2.0 (Simao et al., 2015) was used with the Sordariomyceta dataset and with species parameters set to the nearest represented species (*Neurospora crassa*). This quality assessment was applied to all genomes from the Ophiostomataceae family used during this work and its results are displayed in Supplementary Table 1. Putative BGCs in the *O. ulmi* W9 and *O. novo-ulmi* H327 genomes were identified with antiSMASH 3.0 (using the genome sequence assembly as input) (Weber et al., 2015) and SMIPS 2016-07-26 (using the predicted proteins as input) (Wolf et al., 2015) algorithms. Previously reported results were also examined (Comeau et al., 2015). Notably, BGCs and their fragments can remain in the genome without generating functional products (Campbell et al., 2012). Some genes of these apparently non-functional BGCs may be subject to pseudogenization, driving the synthesis of truncated products (Campbell et al., 2012). To validate the backbone gene of these predicted BGCs and obtain insight about possible non-functional BGCs, we analyzed RNA-seq data from *O. novo-ulmi* H327 (NCBI’s BioProject access: PRJNA325932) (Nigg et al., 2015). Briefly, in the experiment conducted by Nigg et al. (2015), *O. novo-ulmi* H327 was grown in three conditions and both fungal lifestyle structures were analyzed: (1) yeast in liquid culture medium; (2) mycelium in static liquid culture medium; and (3) mycelium in solid culture medium. The detailed RNA-seq experimental procedure, sequencing, data management, and statistics have been previously described (Nigg et al., 2015). Expression data for each gene and differentially expressed genes were extracted from supplementary material (http://www.g3journal.org/content/5/11/2487.supplemental; Supplementary Tables S4–S6). Furthermore, the borders of each cluster that were initially delimited based on antiSMASH 3.0 prediction were subsequently confirmed using CASSIS 2016-10-20 (Wolf et al., 2015) or compared with conserved orthologous gene clusters of other filamentous fungi species using MultiGeneBlast 1.1.14 (Medema et al., 2013).

The conservation of predicted BGCs among Ophiostomataceae species (*Ophiostoma piceae* strain UAMH 11346, *Sporothrix schenckii* strain 1099-18, *Sporothrix brasiliensis* strain 5110, *Sporothrix pallida* strain SPA8, *Grosmannia clavigera* strain kw1407, *Graphilbum fragrans* strain CBS 138720, *Leptographium lundbergii* strain CBS 138716, and *Leptographium procerum* strain CMW34542) was assessed using MultiGeneBlast 1.1.14 (analyses of genomic loci with predicted proteins) (Medema et al., 2013), based primarily on backbone gene conservation (amino acid sequences; *e*-value < 1 × 10^-5^, query coverage ≥ 50%, and identity ≥ 50%). The same approach was used for vicinity analysis of the OpPKS8 locus. Afterward, BLASTP^1^ (non-redundant protein sequence database) (Altschul et al., 1990) was used to search for and curate orthologous gene clusters among other genomes of filamentous fungi and to select putative orthologous backbone genes for the phylogenetic analysis of the OpPKS8 backbone gene (OphioH327gp7312) (three cutoffs were defined: *e*-value < 1 × 10^-5^, query coverage ≥ 50%, and identity ≥ 45%). To further confirm the orthology of collected genes, amino acid sequences of all proteins per genome of species harboring putative OpPKS8 backbone gene orthologs (13 species with annotated genomes) were subjected to OrthoMCL clustering (clustering thresholds: *e*-value < 1 ×-10^-5^ and identity ≥ 30%) (Supplementary Table 1) (Li et al., 2003). Additionally, several fungal genome sequences have been deposited at NCBI as raw or incomplete assemblies from projects that generally employed whole genome shotgun (WGS) strategies. These projects do not have predicted proteins/genes/mRNAs deposited at NCBI, and are therefore inaccessible through routine BLASTP against the non-redundant protein sequences (nr) database and BLASTN against the nucleotide collection (nr/nt) database. Thus, initial screening using BLASTN against the WGS database revealed several unannotated genomes (*Purpureocillium lilacinum* strain TERIBC 1, *Biatriospora mackinnonii* strain E5202H, *Phialocephala scopiformis* strain CBS 120377, *Cairneyella variabilis* strain VPRI 42388, *Shiraia* spp. strain Slf14, *Endocalyx cinctus* strain JCM 7946, *Paecilomyces hepiali* strain FENG, *Pyrenochaeta lycopersici* strain CRA-PAV ER-1211, *Talaromyces purpureogenus* strain MYA38, *Aureobasidium pullulans* strain AY4, and *Scedosporium aurantiacum* strain WM 09.24) (Chan et al., 2012; Vandeputte et al., 2014; Yang et al., 2014; Malapi-Wight et al., 2015; Pérez-Bercoff et al., 2015; Walker et al., 2016; Yu et al., 2016) that harbor putative OpPKS8 backbone gene orthologs. The content of these genomes was downloaded and further explored for the presence of OpPKS8 gene cluster orthologs (through MultiGeneBlast 1.1.14), which were annotated using FGENESH (gene-finding parameters for the closest species presented in the algorithm) (Solovyev et al., 2006). Corresponding amino acid sequences were aligned with the predicted OpPKS8 backbone gene products from *O. ulmi* W9 and *O. novo-ulmi* H327. Genes that satisfied the previously fixed cutoffs were included in further analyses.

### Phylogenetic Analyses

Since the phylogeny of PKS genes can be problematic, particularly for ortholog definition, we included collected entries comprising putative orthologs of the OpPKS8 backbone gene and all characterized PKS from MIBiG, a database of characterized BGCs (Medema et al., 2015), for phylogenetic reconstruction (Sbaraini et al., 2016). This tree helps to determine if the collected entries are true orthologs or artifacts. The amino acid alignment was built using PRANK v.100701, without manual curation (Loytynoja and Goldman, 2010). The best-fit evolutionary model was estimated using Prottest 3.4 (Darriba et al., 2011) (Supplementary Table 2), and phylogenetic reconstruction was conducted using two different methods (both results are available in Supplementary Figure 1): Maximum Parsimony, as implemented in MEGA 6 (Tamura et al., 2013) with 1,000 bootstrap replicates, and Bayesian Inference using MrBayes (Altekar et al., 2004; Ayres et al., 2012; Ronquist et al., 2012). The latter method was executed for up to 10^7^ Markov chain Monte Carlo (MCMC) generations (sampled every 100 steps), applying an average standard deviation of split frequencies < 0.01, calculated every 1000th generation, as the main convergence criterion. The first 25% of samples, corresponding to the initial stages in MCMC sampling where likelihood values increase rapidly, were discarded as burn-in, summarizing parameters and trees afterward. After this phylogenetic confirmation of orthology, amino acid sequences (BLAST-collected entries for the OpPKS8 backbone gene product in addition to FGENESH-annotated entries) were manually trimmed and only the keto-synthase, acyltransferase, and dehydratase protein domains sequences were aligned. Alignment reliability was assessed with GUIDANCE 2.0 (alignment is shown in Supplementary Data Sheet 1), using PRANK (Loytynoja and Goldman, 2010) for sequence alignment with 100 bootstrap replicates and variable gap penalties and a GUIDANCE2 score cutoff of 0.90 for site removal (Landan and Graur, 2008). The best-fit evolutionary model was estimated as described above, and trees for this dataset were constructed using PhyML 3.1 (Guindon et al., 2010) with aLRT SH-like (approximate likelihood ratio test Shimodaira–Hasegawa) branch support estimation (Anisimova and Gascuel, 2006; Anisimova et al., 2011) and MrBayes, as described above.

A species tree based on 18S ribosomal RNA gene (partial), internal transcribed spacer 1, 5.8S ribosomal RNA gene, internal transcribed spacer 2, and 28S ribosomal RNA gene (partial) sequences was constructed to clarify the relationships between the Ophiostomataceae species used in comparative genomic analysis (Carlier et al., 2006; Romon et al., 2014). Additionally, a *Neurospora crassa* entry was added as an outgroup. The best-fit evolutionary model was assessed using jModeltest 2.1.9 (Darriba et al., 2012), and a phylogenetic tree was constructed using PhyML 3.1 with 100 bootstrap replicates (alignment shown in Supplementary Data Sheet 2). A logical diagram describing the step-by-step process and the connections between methodologies applied in this section is shown in Supplementary Figure 2.

## Results

### Predictions of Secondary Metabolite Biosynthetic Gene Clusters

**FIGURE 1:**
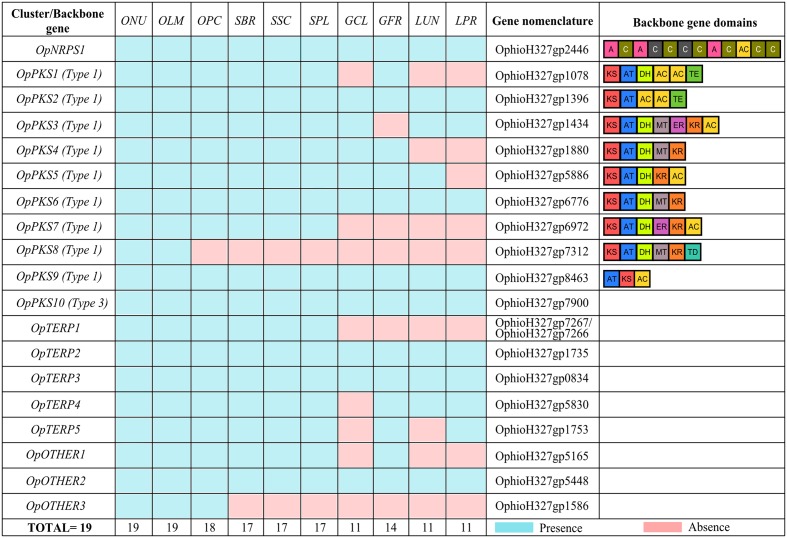
Biosynthetic gene cluster (BGC) conservation in the Ophiostomataceae family. *O. novo-ulmi* (ONU), *O. ulmi* (OLM), *O. piceae* (OPC), *S. brasiliensis* (SBR), *S. schenckii* (SSC), *S. pallida* (SPL), *G. clavigera* (GCL), *G. fragrans* (GFR), *L. lundbergii* (LUN), and *L. procerum* (LPR). Nomenclature adapted from Comeau et al. (2015) and domains for PKS and NRPS backbone gene are listed. Adenylation (A), Condensation (C), Peptidyl Carrier Protein (PCP), Keto-synthase (KS), Acyltransferase (AT), Acyl Carrier Protein (AC), Dehydrogenase (DH), Enoylreductase (ER), Ketoreductase (KR), Methyltransferase O- or C- (MT), Thioesterase (TE), Thioester reductase (TD). The conservation of predicted BGCs among Ophiostomataceae species was assessed using MultiGeneBlast 1.1.14 (analyses of genomic loci with predicted proteins) (Medema et al., 2013), based primarily on backbone gene conservation (*e*-value < 1 × 10^-5^, query coverage ≥ 50%, and identity ≥ 50%).

A genome survey predicted 19 putative BGCs/backbone genes in *O. ulmi* W9 and *O. novo-ulmi* H327 genomes. Interestingly, the metabolic potential of *O. ulmi* W9 and *O. novo-ulmi* H327 seemed identical. All BGCs found in *O. ulmi* W9 were also found in *O. novo-ulmi* H327, and vice versa, and included 1 NRPS, 10 PKS, 5 terpenes (TERP), and three BGCs that were classified as “OTHER” by antiSMASH, a generic class of gene clusters grouping BGCs with unusual backbone genes (e.g., NRPS-like and PKS-like) (**Figure 1** and Supplementary Data Sheet 3).

To validate the predicted backbone genes of DED pathogens and obtain insight about possible non-functional BGCs, RNA-seq data from *O. novo-ulmi* H327 was analyzed as it was expected that possible non-functional BGCs would not have mapped reads. All of the predicted backbone genes exhibited mapped reads in at least one of the three conditions (yeast in liquid culture medium, mycelium in static liquid culture medium, or mycelium in solid culture medium), indicating gene functionality (Supplementary Table 3). Additionally, the expression of OpPKS10 BGC and OpOTHER1 BGC appeared to be regulated by fungal lifestyle structures, since the backbone genes (OphioH327g7900 and OphioH327g5165) of these clusters are up-regulated in mycelium (mycelium in static liquid culture medium × yeast in liquid culture medium [logFC: 6.71; FDR ≤ 1%] and mycelium in solid culture medium × yeast in liquid culture medium [logFC: 5.46; FDR ≤ 1%]) and yeast (yeast in liquid culture medium × mycelium in static liquid culture medium [logFC: 1.2; FDR ≤ 1%] and yeast in liquid culture medium × mycelium in solid culture medium [logFC: 1.9; FDR ≤ 1%]), respectively (Nigg et al., 2015) (Supplementary Table 3).

### Conservation of BGCs in the Ophiostomataceae Family

To identify SM gene clusters unique to DED pathogens, we assessed BGC conservation among eight Ophiostomataceae family species. The proximity of these species to *O. ulmi* W9 and *O. novo-ulmi* H327 was confirmed by phylogeny (Supplementary Figure 3), and completeness and quality of genome sequence assembly/predicted proteins was assessed using BUSCO v2 (all genomes had >89% of the benchmarking universal single-copy orthologs expected to be conserved in Sordariomycetes; Supplementary Table 1). The majority of BGCs (>89%) found in DED pathogens were well-conserved in the saprophytic *O. piceae* UAMH 11346 (18 conserved BGCs) and in species from the closely related *Sporothrix* genus (17 conserved BGCs in *S. schenckii* 1099-18, *S. brasiliensis* 5110, and *S. pallida* SPA8) (**Figure 1**). Interestingly, more than 50% of the predicted BGCs detected in DED pathogens were also conserved in more distantly related species such as *G. fragrans* CBS 138720 (14 conserved BGCs), *G. clavigera* kw1407 (11 conserved BGCs), *L. lundbergii* CBS 138716 (11 conserved BGCs), and *L. procerum* CMW34542 (11 conserved BGCs) (**Figure 1**). These results indicate that the SM potential is remarkably similar between Ophiostomataceae family members. Notably, two BGCs were found only in the *Ophiostoma* genus: OpOTHER3 (OphioH327gp1586) was identified in *O. piceae* UAMH 11346, *O. ulmi* W9, and *O. novo-ulmi* H327, while OpPKS8 is exclusive to DED pathogens. To further confirm the absence of OpPKS8 in *O. piceae* UAMH 11346, the vicinities of the OpPKS8 locus were explored. The results confirmed the absence of the OpPKS8 gene cluster in *O. piceae* UAMH 11346, as several genes in the vicinity were conserved despite the absence of OpPKS8 (Supplementary Figure 4).

### Comparative Genomic Analyses of BGCs and Phylogeny

Considering that no putative BGCs have been functionally characterized in DED pathogens to date, we employed comparative genomics to determine the likely final products of these BGCs. These comparisons revealed three interesting BGCs that exhibited previously characterized orthologous gene clusters in other species and which were putatively linked with the biosynthesis of a fujikurin-like compound (OpPKS8), a siderophore compound (OpNRPS1), and pyrones and resorcylic acids (OpPKS10, a type III PKS).

Orthologs for the OpPKS10 backbone gene were found in all evaluated Ophiostomataceae species (**Figure 1**). The predicted backbone gene protein for this cluster (OphioH327gp7900) exhibited 63% identity with the protein sequence of a type III PKS involved in the biosynthesis of triketide pyrones, tetraketide pyrones, tetraketide resorcylic acids, and pentaketide resorcylic acids characterized in *N. crassa* (Goyal et al., 2008; Rubin-Pitel et al., 2008). *N. crassa* and the Ophiostomataceae family species are grouped in the same class, which may indicate that proteins with similar functions to those of *N. crassa* could represent the final product of the OpPKS10 backbone gene, and that these similar proteins could act in the biosynthesis of similar metabolites. Synteny comparison between the type III PKS locus in DED pathogens and in *N. crassa* did not reveal extensive conservation (Supplementary Figure 4), suggesting that it might not be grouped in a biosynthetic cluster. Reinforcing this hypothesis, CASSIS was unable to discriminate homologous regulatory sequences between type III PKS of DED pathogens and neighboring genes.

OpNRPS1 BGC shows similarity to characterized NRPS involved in siderophore biosynthesis (Supplementary Figure 4). Iron is an essential micronutrient for almost all organisms, including fungi (Silva-Bailao et al., 2014). Thus, these microorganisms have developed highly efficient iron-acquisition systems. Siderophores are chelators synthesized by microbes to sequester iron (Haas, 2014). Siderophore biosynthesis has been widely explored in model fungi such as *Aspergillus fumigatus*, *Paracoccidioides brasiliensis*, and *Metarhizium anisopliae* (Haas, 2014; Silva-Bailao et al., 2014; Giuliano Garisto Donzelli et al., 2015; Sbaraini et al., 2016). In these model species, siderophores are essential for successful infection (Schrettl et al., 2007; Giuliano Garisto Donzelli et al., 2015). Primary screening (BLASTP cutoffs: *e*-value < 1 × 10^-5^, query coverage ≥ 50%, and identity ≥ 45%; searching against characterized gene clusters/backbone genes deposited in MIBiG) did not reveal genes for siderophore biosynthesis in DED pathogens. However, in view of the importance of siderophores for the infection of several fungal species, we deepened our analysis by decreasing the previously fixed cut-offs (BLASTP cut-offs: *e*-value < 1 × 10^-5^, query coverage ≥ 50%, and identity ≥ 30%). Additionally, since siderophore biosynthesis in filamentous fungi is usually catalyzed by NRPS and DED pathogens have a single NRPS gene cluster (OpNRPS1), we focused on this BGC. Secondary screening revealed that the protein sequence of the OpNRPS1 backbone gene from *O. ulmi* W9 exhibited 38% identity with the backbone gene of the intracellular siderophore ferricrocin (KFG82816.1), which has been characterized in *M. robertsii* and is conserved in *M. anisopliae* (Giuliano Garisto Donzelli et al., 2015; Sbaraini et al., 2016). Additional analysis of OpNRPS1 gene cluster revealed an L-ornithine N5-oxygenase gene (OphioH327gp2447) in the BGC (Supplementary Figure 4). The product of this gene exhibited 48% identity with an L-ornithine N5-oxygenase (SidA) from *Aspergillus nidulans* (Eisendle et al., 2003). It has been hypothesized that, in *A. nidulans*, the first biosynthetic step for both intra- and extracelluar siderophores is catalyzed by ornithine oxygenates such as SidA (Plattner and Diekmann, 1994). In agreement with this hypothesis, mutational inactivation of SidA abolishes siderophore biosynthesis, completely attenuating the virulence of *A. nidulans* to mice (Eisendle et al., 2003). This clustering of the ferricrocin ortholog backbone gene and the L-ornithine N5-oxygenase gene suggests, therefore, that DED pathogens utilize a siderophore biosynthesis pathway that is similar to other model fungi. Furthermore, we hypothesize that the products of OpNRPS1 BGC modulate iron dynamics in DED pathogens.

**FIGURE 2:**
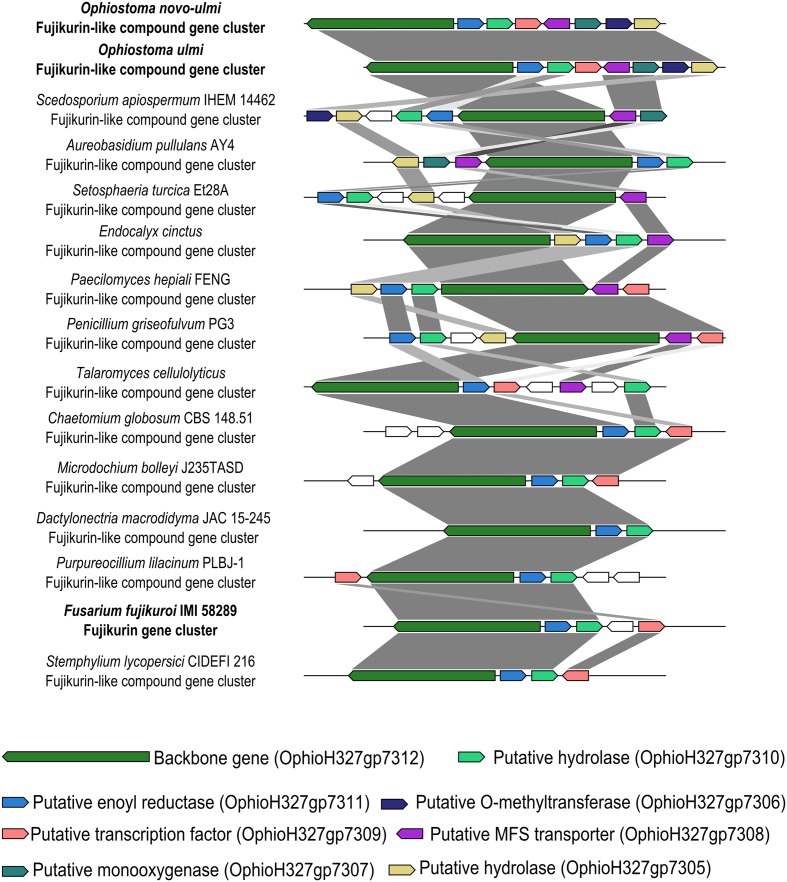
Putative fujikurin and fujikurin-like compound cluster (OpPKS8) conservation and synteny. The OpPKS8 cluster from DED pathogens resembled the characterized fujikurin cluster from *F. fujikuroi* IMI 58289 (36–68% identity in protein-by-protein comparisons of BGC constituent genes), and putative BGCs from *Scedosporium apiospermum* (66–84% identity in protein-by-protein comparisons of BGC constituent genes), *Aureobasidium pullulans* (58–80% in protein-by-protein comparisons of BGC constituent genes), and *Setosphaeria turcica* (66–70% identity in protein-by-protein comparisons of BGC constituent genes). The conservation between OpPKS8 and these putative BGCs from *S. apiospermum*, *A. pullulans*, and *S. turcica* helped to define the boundaries from OpPKS8.

**FIGURE 3:**
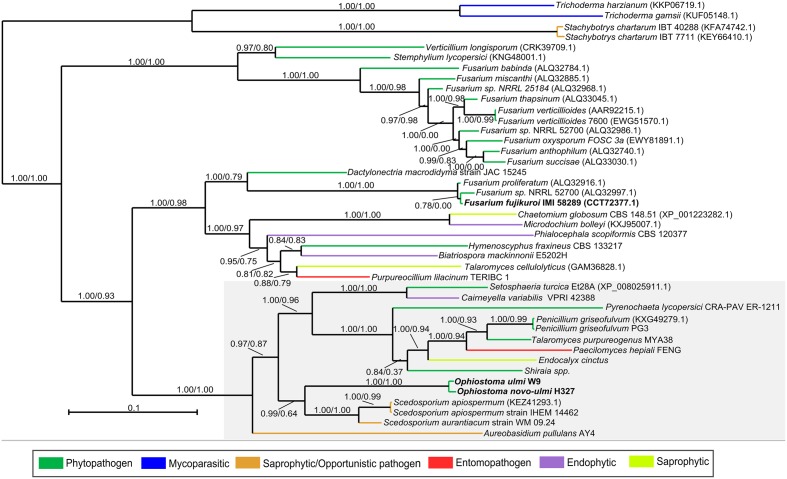
Fujikurin-like compound BGC (OpPKS8). Phylogenetic analyses were performed using Maximum-likelihood and Bayesian Inference, based on the ketosynthase, acyltransferase, and dehydrogenase domains of the fujikurin-like backbone gene of DED pathogens and orthologous sequences exhibited by several fungi. The orthologous sequences were classified according to fungal lifestyle trait, represented by different colors. The Bayesian tree is displayed and branch support values (aLRT SH-like supports and Bayesian posterior probability) are associated with nodes. The Bayesian inference ran for 35,000 generations. DED pathogens and *F. fujikuroi* IMI 58289 harboring the fujikurin and fujikurin-like gene clusters are highlighted in bold. BGCs closely related to the OpPKS8 gene cluster are shaded gray. Notably, species that harbor these BGCs closely related to the OpPKS8 gene cluster have a plant-associated trait.

The OpPKS8 gene cluster matched the characterized fujikurin BGC from *Fusarium fujikuroi* IMI 58289, and showed considerable identity (36–68% identity in protein-by-protein comparisons of BGC constituent genes), albeit a different cluster configuration (**Figure 2**) (Wiemann et al., 2013; von Bargen et al., 2015). von Bargen et al. (2015) have proposed that the fujikurin gene cluster (also called PKS19) of *F. fujikuroi* IMI 58289 is composed of five genes: a highly reducing PKS (CCT72377) exhibiting 58% identity with the OpPKS8 backbone gene (OphioH327gp7312); a protein with a predicted enoyl reductase domain (CCT72378) exhibiting 63% identity with the ortholog present in OpPKS8 BGC (OphioH327gp7311); a protein with a predicted hydrolase domain (CCT72379) exhibiting 50% identity with the ortholog present in OpPKS8 BGC (OphioH327gp7310); a putative transcription factor (CCT72381) exhibiting 36% identity with the ortholog present in OpPKS8 BGC (OphioH327gp7309); and a putative transporter (CCT72380) that does not have an ortholog in OpPKS8 BGC (von Bargen et al., 2015). Thus, the basic components of the fujikurin biosynthetic route appear to be conserved in DED pathogens. Additionally, comparative genomics and phylogeny have revealed that the OpPKS8 gene cluster is phylogenetically related to putative BGCs found in *Scedosporium apiospermum* IHEM 14462 (66–84% identity in protein-by-protein comparisons of constituent genes of these BGCs), *Aureobasidium pullulans* AY4 (58–80% identity) and *Setosphaeria turcica* Et28A (66–70% identity) (**Figures 2**, **3**). Some genes conserved between the OpPKS8 gene cluster and the BGCs from *S. apiospermum*, *A. pullulans*, and *S. turcica* coded for proteins with known implications in SM biosynthesis. This led to the hypothesis that other genes may be involved in the biosynthesis of the final compound of the OpPKS8 gene cluster (unconserved genes in *F. fujikuroi*), namely, a fujikurin-like compound. In addition to the previously cited genes, the proposed OpPKS8 BGC contains the following: a putative *O*-methyltransferase protein (OphioH327gp7306) which exhibits 77% identity with an ortholog from *S. apiospermum* KEZ41288; absent in *A. pullulans* and *S. turcica*); a protein with a predicted hydrolase domain (OphioH327gp7305) exhibiting 79% identity with an *S. apiospermum* ortholog (KEZ41289); a putative MFS transporter protein (OphioH327gp7308) 75% identical to an ortholog from *S. apiospermum* (KEZ41294); and a putative monooxygenase protein (OphioH327gp7307) exhibiting 66% identity with an ortholog in *S. apiospermum* (KEZ41295; absent in *S. turcica*) (**Figure 2**).

Moreover, it is important to note that *S. apiospermum* belongs to a different order (Microascales), and that *A. pullulans* and *S. turcica* belong to a different class (Dothideomycetes), and are therefore considerably distant from DED pathogens (Ophiostomales, Sordariomycetes). Evolutionary distances are best displayed in the schematic Ascomycota tree of life based on the work of Schoch et al. (2009) (Supplementary Figure 5), which contrasts with the OpPKS8 backbone gene tree (**Figure 3**) (Schoch et al., 2009). Additionally, the OpPKS8 gene cluster is the only BGC exclusively found in DED pathogens (among the evaluated species), raising the possibility that HGT events could have shaped the evolution of OpPKS8 orthologs. Alternatively, an ancestor of the Sordariomycetes and Dothideomycetes classes may have contained a fujikurin-like compound gene cluster, which remained in the genome of some species. This hypothesis, however, would have required the loss of constituent genes across several Sordariomycetes and Dothideomycetes species. Additionally, species from Leotiomycetes (*Hymenoscyphus fraxineus*, *Cairneyella variabilis*, and *Phialocephala scopiformis*) and Eurotiomycetes (*Talaromyces cellulolyticus*, *Talaromyces purpureogenus*, and *Penicillium griseofulvum*) classes also have orthologs, making this vertical descent hypothesis unlikely.

Elucidation of the structure of fujikurin has revealed that this compound was previously described as CR377, an SM with antifungal activity isolated from an endophytic *Fusarium* species (Brady and Clardy, 2000). Furthermore, the *F. fujikuroi* IMI 58289 BGC (PKS19) involved in fujikurin production was up-regulated during the infection of rice plants, highlighting a possible role of this metabolite as a phytopathogenic virulence determinant (Wiemann et al., 2013). We sought to analyze the lifestyles (pathogenic or not) of those fungi harboring OpPKS8 backbone gene orthologs. Although cluster configuration changes in these species (**Figure 2**), lifestyle analyses could aid in the prediction of a role for the metabolic product of the OpPKS8 BGC. We were particularly interested in whether other phytopathogenic fungi harbor fujikurin or fujikurin-like compound gene clusters, in view of the putative role of fujikurin in the infection of *Fusarium* species. Regarding BGCs closely related to OpPKS8 (shaded in gray in **Figure 3**), five gene clusters have been found in species with a known phytopathogenic trait; three in widely known opportunistic pathogens (although both *A. pullulans* and *Scedosporium* spp. are associated with plants); one in *Endocalyx cinctus* (a poorly documented species linked with saprophytic growth in dead palms); one in the ericoid mycorrhizal fungus *Cairneyella variabilis*; and one in *Paecilomyces hepiali* (a species with nematophagous/entomopathogenic traits that may be associated with plants). Regarding BGCs more distantly related to the OpPKS8 gene cluster: 16 have been found in phytopathogens, with several *Fusarium* representatives; three in endophytic fungi species; two in saprophytic species; two in opportunistic pathogens; two in widely known mycopathogens; and one in a species with nematophagous/entomopathogenic traits (**Figure 3**). Clearly, several species that possess orthologous genes have a plant-associated lifestyle, leading us to speculate that fujikurin-like SMs allow several fungal species to interact with plants, not only as phytopatogens or endophytes but also in a nematophagous/entomopathogenic or opportunistic manner.

## Discussion

Comparative genomics have provided important insights into the evolution of fungal pathogens. Several fungal genomes have been sequenced in recent years, including multiple Ophiostomataceae family members, and the publication of this data enables deep comparisons to highlight putative virulence determinants. This has allowed relevant analyses, in view of a singular characteristic of different pathogenic traits in close family members (e.g., phytopathogenic and *Sporothrix* mammalian pathogenic fungi), enabling the identification of virulence determinants linked with the phytopathogenic trait (i.e., genes not conserved in *Sporothrix* spp.). Additionally, as the Ophiostomataceae family harbors several phytopathogenic fungi, a putative virulence determinant may be widespread in several species.

Secondary metabolites are important virulence determinants for microorganisms. These molecules are produced to circumvent host defenses and ensure the success of these organisms in the environment (Keller, 2015). We explored the SM potential of DED pathogens and identified three interesting BGCs: OpNRPS1, OpPKS8, and OpPKS10.

Our analysis has putatively linked the OpPKS10 backbone gene, a type III PKS, with a similar gene in *N. crassa*. Its products are structurally simple enzymes, despite producing a wide array of compounds such as chalcones, pyrones, acridones, phloroglucinols, stilbenes, and resorcinolic lipids (Hashimoto et al., 2014). Although type III PKSs have been widely explored in bacteria and plants, type III PKSs were only recently identified and characterized in fungal species such as *N. crassa*, *Aspergillus oryzae*, *Aspergillus niger*, and *Botrytis cinerea* (Hashimoto et al., 2014). In *N. crassa*, for instance, the PKS utilizes long-chain fatty acyl-CoAs as starter units for the biosynthesis of several compounds, its product being dependent on the length of the starter unit (Goyal et al., 2008; Rubin-Pitel et al., 2008). A similar feature of all characterized fungal type III PKSs is pyrone biosynthesis, and it is tempting to suggest that the product of OpPKS10 is involved in this type of synthesis as well. Interestingly, the OpPKS10 backbone gene is up-regulated in mycelium growth, suggesting that its putative products are regulated by fungal lifestyle (Supplementary Table 3) (Nigg et al., 2015). A mycelium phase is essential for invasion of uninfected xylem vessels and for posterior saprophytic growth within moribund trees, and the products of OpPKS10 may be of significant importance during this growth phase.

The OpNRPS1 gene cluster was putatively linked with siderophore biosynthesis. Siderophores are interesting compounds that bind iron, which is fundamental to several metalloprotein-dependent pathways for oxygen transport and storage, electron transfer, and substrate oxidation and reduction (Kurth et al., 2016). Thus, we hypothesize that siderophores play an important role in DED pathogen lifecycle and infection, as in other filamentous fungi. However, while fungi such as *A. fumigatus* and *M. anisopliae*, and phytopathogens such as *Cochliobolus heterostrophus* have two pathways for siderophore biosynthesis (i.e., intra- and extracellular) (Schrettl et al., 2007; Condon et al., 2014; Haas, 2014; Giuliano Garisto Donzelli et al., 2015), we found only one putative intracellular siderophore BGC in DED pathogens. The apparent absence of extracellular siderophores is somewhat surprising, and further investigation is clearly needed to explore iron metabolism in DED pathogens. For example, other iron-excavating systems may be active in these fungal species for the maintenance of iron homeostasis. Furthermore, the absence of this extracellular siderophore BGC could be a strategy to evade host immunity, since these compounds are thought to trigger immunity in plants; therefore, the absence of extracellular siderophores could be advantageous (Aznar and Dellagi, 2015).

For the OpPKS8 gene cluster (a putative fujikurin-like compound gene cluster), we performed individual phylogeny beyond the comparative genomic analysis. This was because this cluster was conserved only in DED pathogens, and absent in the genomes of other Ophiostomataceae family members analyzed, especially that of *O. piceae* UAMH 11346. Both comparative genomic and phylogenetic analyses revealed that the OpPKS8 gene cluster may have been acquired by a HGT event, and its orthologs are conserved in plant-related fungi. Despite lateral transfer, closely orthologous BGCs are found in species distantly related to the Ophiostomatales order (Supplementary Figure 5). In fact, extensive HGT events have been reported in several pathogens and could potentially be a way to increase the range of susceptible hosts, as well as to adapt to new environments. These incidents could have increased as a result of the intensifying global trade and traffic of host plant species infected by different pathogens (Mehrabi et al., 2011). Interestingly, HGT events involving DED pathogens have already been reported. Fungi from the *Geosmithia* genus (Hypocreales order), isolated from *Ulmus minor* trees infected with DED pathogens, harbor fragments from a hydrophobin cerato-ulmin gene from *O. novo-ulmi*. Both *Ophiostoma* and *Geosmithia* genera occupy similar habitats and frequently co-occur, which was suggested as a favorable condition for the transfer of genetic material (Bettini et al., 2014). A similar case can be suggested for the evolution and diversification of fujikurin-like compound BGCs. Sympatry may have facilitated HGT events, since these SM gene clusters are predominantly found in phytopathogenic and endophytic fungi, in addition to some nematophagous/entomopathogenic, saprophytic, mycopathogenic, and opportunistic pathogenic fungi. Moreover, the OpPKS8 backbone gene (OphioH327gp7312) has mapped reads in all three tested conditions (yeast in liquid culture medium; mycelium in static liquid culture medium; and mycelium in solid culture medium) by Nigg et al. (2015) and is therefore indicative of full functionality, since this BGC may generate functional transcripts and subsequent proteins which would help to catalyze the biosynthesis of natural products.

However, some care must be taken in relation to OpPKS8 BGC distribution and its presence in *Ophiostoma* genus and Ophiostomataceae family species. Although our results are applicable to two significant strains of two DED-related species [especially, *O. novo-ulmi* H327, described as a highly aggressive pathogen (Et-Touil et al., 1999)] future research based on PCR validation of other field strains or genome sequencing should be carried out in order to evaluate whether the OpPKS8 gene cluster is species-specific or strain-specific, as well as to better understand the distribution of OpPKS8 within the *Ophiostoma* genus. In this sense, during the submission of our work, the genome of the spruce pathogen *Ophiostoma bicolor* strain ZLVG358 was published (Lah et al., 2017). This genome exhibits an ortholog (obic_04723) for the OpPKS8 backbone gene (65% identity between proteins), indicating that other phytopathogenic species from the *Ophiostoma* genus may harbor OpPKS8 orthologs.

The SM potential of DED pathogens was meticulously analyzed and yielded insights into aspects of genome organization of BGCs, their expression, and their possible role as virulence determinants. Although the importance of OpPKS10, OpNRPS1, and mainly OpPKS8 gene clusters during DED pathogenic infection requires further confirmation using wet lab techniques, our findings are important for future research. Fujikurins and fujikurin-like compounds can play significant roles in fungal-plant interactions in several models, including several economically important phytopathogenic fungi, in addition to presenting horizontally transferred origins. Additionally, we anticipate that other putative virulence determinants can be found in DED pathogens and Ophiostomaceae family members using comparative genomic approaches, as this family is rich in pathogenic traits. Other relevant genetic characteristics could be explored in both *Sporothrix* and *Ophiostoma* genera in addition to genetic singularities that may be important virulence determinants.

## Author Contributions

Conceived and designed the experiments: NS, TC, MV, and AS. BGC curation and comparative analyses: NS, ÂJ, RG, and TC. Phylogenetic analyses: NS, FA, and CT. Contributed reagents/materials/analysis tools: MV, AV, CS, and AS. Wrote the manuscript: NS, FA, CT, ÂJ, RG, and AS. All authors read and approved the final manuscript.

## Conflict of Interest Statement

The authors declare that the research was conducted in the absence of any commercial or financial relationships that could be construed as a potential conflict of interest.

## Footnotes

1 http://blast.ncbi.nlm.nih.gov/Blast.cgi
