# Supplementary material for: Genome-Wide Analysis of Secondary Metabolite Gene Clusters in Ophiostoma ulmi and Ophiostoma novo-ulmi Reveals a Fujikurin-Like Gene Cluster with a Putative Role in Infection
Source: Front Microbiol. 2017 Jun 13;8:1063. doi: 10.3389/fmicb.2017.01063 (PMC5468452; doi:10.3389/fmicb.2017.01063)
Supplement: Supplementary file 1 [file Table_1.DOCX]

***Supplementary Table 1***

**Genome-wide analysis of secondary metabolite gene clusters in O*phiostoma* *ulmi* and *Ophiostoma novo-ulmi* reveals a fujikurin-like gene cluster with a putative role in infection**

**Nicolau Sbaraini ^1, 2^, Fábio Carrer Andreis ^1, 2^, Claudia Elizabeth Thompson ^1, 2, 3^, Rafael Lucas Muniz Guedes ^1, 3^, Ângela Junges ^2^, Thais Campos ^2^, Charley Christian Staats^1, 2^, Marilene Henning Vainstein ^1, 2^, Ana Tereza Ribeiro de Vasconcelos ^1, 3^, Augusto Schrank ^1, 2,*^.**

*** Correspondence:**Augusto Schrank
[aschrank@cbiot.ufrgs.br](mailto:aschrank@cbiot.ufrgs.br)

**Table 1: Fungal genomes used in this work.**

| Genome | NCBI’s Bioproject Number | Genbank Assembly Accession Number | Nucleotide Sequence Accession Number |
| --- | --- | --- | --- |
|  |  |  |  |
| Genomes from Ophiostomataceae family species |  |  |  |
|  |  |  |  |
| *Ophiostoma novo-ulmi* H327 | PRJNA173023 | GCA_000317715.1 | AMZD00000000 |
| *Ophiostoma ulmi* W9* |  |  |  |
| *Ophiostoma piceae* UAMH 11346 | PRJNA182071 | GCA_000410735.1 | AQHS00000000 |
| *Sporothrix brasiliensis* 5110 | PRJNA218075 | GCA_000820605.1 | AWTV00000000 |
| *Sporothrix schenckii* 1099-18 | PRJNA218070 | GCA_000961545.1 | AXCR00000000 |
| *Sporothrix pallida* SPA8 | PRJNA248334 | GCA_000710705.2 | JNEX00000000 |
| *Grosmannia clavigera* kw1407 | PRJNA39837 | GCA_000143105.2 | ACXQ00000000 |
| *Graphilbum fragrans* CBS 138720 | PRJNA298958 | GCA_001513895.1 | LLKO00000000 |
| *Leptographium lundbergii* CBS138716 | PRJNA284246 | GCA_001455505.1 | LDEF00000000 |
| *Leptographium procerum* CMW34542 | PRJNA262457 | GCA_000806385.1 | JRUC00000000 |
|  |  |  |  |
| Unannotated genomes |  |  |  |
|  |  |  |  |
| *Dactylonectria macrodidyma* JAC 15-245 | PRJNA274176 | GCA_000935225.1 | JYGD00000000 |
| *Hymenoscyphus fraxineus* CBS 133217 | PRJNA297734 | GCA_001414365.1 | LLCC00000000 |
| *Purpureocillium lilacinum* TERIBC 1 | PRJNA284314 | GCA_001468795.1 | LOFA00000000 |
| *Biatriospora mackinnonii* E5202H | PRJNA187080 | GCA_001007845.1 | JGVQ00000000 |
| *Phialocephala scopiformis* CBS 120377 | PRJNA262390 | GCF_001500285.1 | LKNI00000000 |
| *Cairneyella variabilis* VPRI 42388 | PRJNA225634 | GCA_001625345.1 | AYLM00000000 |
| *Shiraia* spp*.* Slf14 | PRJNA222884 | GCA_000498155.1 | AXZN00000000 |
| *Endocalyx cinctus* JCM 7946 | PRJDB3783 | GCA_001600455.1 | BCKC00000000 |
| *Paecilomyces hepiali* FENG | PRJNA301665 | GCA_001455915.2 | LNDK00000000 |
| *Pyrenochaeta lycopersici* CRA-PAV_ER 1211 | PRJNA202288 | GCA_000601435.1 | ASRS00000000 |
| *Talaromyces purpureogenus* MYA-38 | PRJNA276974 | GCA_001270325.1 | LIAB00000000 |
| *Aureobasidium pullulans* AY4 | PRJNA171022 | GCA_000294735.1 | AMCU00000000 |
| *Scedosporium aurantiacum* WM 09.24 | PRJNA261653 | GCA_000812075.1 | JUDQ00000000 |
|  |  |  |  |
| Genomes used for OrthoMCL clustering |  |  |  |
|  |  |  |  |
| *Chaetomium globosum* CBS 148.51 | PRJNA12795 | GCA_000143365.1 | AAFU00000000 |
| *Fusarium fujikuroi* IMI 58289 | PRJEB185 | GCA_900079805.1 |  |
| *Fusarium verticillioides* 7600 | PRJNA245136 | GCF_000149555.1 | AAIM00000000 |
| *Microdochium bolleyi* J235TASD1 | PRJNA308023 | GCA_001566295.1 | LSSP00000000 |
| *Penicillium griseofulvum* PG3 | PRJNA289974 | GCA_001561935.1 | LHQR00000000 |
| *Scedosporium apiospermum*  IHEM 14462 | PRJNA244532 | GCF_000732125.1 | JOWA00000000 |
| *Stachybotrys chartarum* IBT 40288 | PRJNA170468 | GCA_000732765.1 | AQPQ00000000 |
| *Stemphylium lycopersici* CIDEFI 216 | PRJNA274742 | GCA_001191545.1 | LGLR00000000 |
| *Setosphaeria turcica* Et28A | PRJNA82947 | GCA_000359705.1 | AIHT00000000 |
| *Talaromyces cellulolyticus* Y-94 | PRJDB3250 | GCA_000829775.1 | BBPS00000000 |
| *Trichoderma gamsii* T6085 | PRJNA252048 | GCA_001481775.1 | JPDN00000000 |
| *Trichoderma harzianum* T6776 | PRJNA252551 | GCA_000988865.1 | JOKZ00000000 |
| *Verticillium longisporum* VL1 | PRJEB9342 | GCA_001268145.1 | CVQH00000000 |
|  |  |  |  |

*Ophiostoma ulmi W9 genome and predicted proteins was downloaded from http://www.moseslab.csb.utoronto.ca/o.ulmi/

**Table 2: Comparison of the primary genome features between DED pathogens and Ophiostomataceae family species.**

| **Feature** | ***ONU*** | ***OLM*** | ***OPC*** | ***SBR*** | ***SSC*** | ***SPL*** | ***GCL*** | ***GFR*** | ***LUN*** | ***LPR*** |
| --- | --- | --- | --- | --- | --- | --- | --- | --- | --- | --- |
|  |  |  |  |  |  |  |  |  |  |  |
| **Strain** | H327 | W9 | UAMH 11346 | 5110 | 1099–18 | SPA8 | kw1407 | CBS138720 | CBS138716 | CMW34542 |
| **Host** | *Ulmus* sp. | *Ulmus* sp. | Saprophytic | Mammals | Mammals | Saprophytic | Coniferous | Coniferous | Coniferous | Coniferous |
| **Sequencing plataform** | 454 | Illumina | Illumina | 454 | 454 | IonTorrent/  Illumina | Sanger/  454/  Illumina | Illumina | Illumina | Illumina |
| **Size (Mbp)** | 31.8 | 31.5 | 33 | 33.2 | 32.4 | 37.8 | 29.8 | 34.2 | 26.6 | 28.6 |
| **Coverage** | 61x | 200x | 735x | 20x | 17x | 50x | 64x | 64x | 70x | 32x |
| **Contigs** | 161 | 3,415 | 381 | 601 | 237 | 432 | 334 | 393 | 735 | 2,747 |
| **Protein-coding genes** | 8,640 | 8,639 | 8,884 | 9,091 | 10,293 | * | 8,314 | * | * | * |
|  |  |  |  |  |  |  |  |  |  |  |

*Genomes without predicted proteins deposited in public databases.

**Table 3: BUSCO results against a subset of 3725 single-copy orthologs putatively conserved in Sordariomycetes**

| **Species** | **Size** | **BUSCO notation assessment results** |
| --- | --- | --- |
| *Ophiostoma novo-ulmi* H327 | 8,640 proteins | C:97.6%[S:97.5%,D:0.1%],F:1.9%,M:0.5% |
| *Ophiostoma ulmi* W9 | 8,639 proteins | C:95.7%[S:95.7%,D:0.0%],F:3.1%,M:1.2% |
| *Ophiostoma piceae* UAMH 11346 | 8,884 proteins | C:96.1%[S:96.1%,D:0.0%],F:2.4%,M:1.5% |
| *Sporothrix brasiliensis* 5110 | 9,091 proteins | C:92.0%[S:92.0%,D:0.0%],F:4.7%,M:3.3% |
| *Sporothrix schenckii* 1099-18 | 10,293 proteins | C:95.3%[S:95.2%,D:0.1%],F:3.2%,M:1.5% |
| *Sporothrix pallida* SPA8 | 37.8 Mbp | C:90.5%[S:90.3%,D:0.2%],F:0.8%,M:8.7% |
| *Grosmannia clavigera* kw1407 | 8,314 proteins | C:91.6%[S:91.5%,D:0.1%],F:4.7%,M:3.7% |
| *Graphilbum fragrans* CBS138720 | 34.2 Mbp | C:94.9%[S:94.1%,D:0.8%],F:2.1%,M:3.0% |
| *Leptographium lundbergii* CBS138716 | 26.6 Mbp | C:96.3%[S:96.2%,D:0.1%],F:1.1%,M:2.6% |
| *Leptographium procerum* CMW34542 | 28.6 Mbp | C:89.5%[S:89.4%,D:0.1%],F:5.0%,M:5.5% |

Complete BUSCOs (C); Complete and single-copy BUSCOs (S); Complete and duplicated BUSCOs (D); Fragmented BUSCOs (F); Missing BUSCOs (M).
