# Supplementary material for: Genome-Wide Analysis of Secondary Metabolite Gene Clusters in Ophiostoma ulmi and Ophiostoma novo-ulmi Reveals a Fujikurin-Like Gene Cluster with a Putative Role in Infection
Source: Front Microbiol. 2017 Jun 13;8:1063. doi: 10.3389/fmicb.2017.01063 (PMC5468452; doi:10.3389/fmicb.2017.01063)
Supplement: Supplementary file 2 [file Table_2.DOCX]

***Supplementary Table 2***

**Genome-wide analysis of secondary metabolite gene clusters in *Ophiostoma ulmi* and *Ophiostoma novo-ulmi* reveals a fujikurin-like gene cluster with a putative role in infection**

**Nicolau Sbaraini ^1, 2^, Fábio Carrer Andreis ^1, 2^, Claudia Elizabeth Thompson ^1, 2, 3^, Rafael Lucas Muniz Guedes ^1, 3^, Ângela Junges ^2^, Thais Campos ^2^, Charley Christian Staats^1, 2^, Marilene Henning Vainstein ^1, 2^, Ana Tereza Ribeiro de Vasconcelos ^1, 3^, Augusto Schrank ^1, 2,*^.**

*** Correspondence:**Augusto Schrank
aschrank@cbiot.ufrgs.br

Best-fit evolutionary models predicted with Prottest 3.4 or jModeltest-2.1.9 for each alignment.

| Gene/Tree | Model |
| --- | --- |
| OpPKS8 | JTT+I+G |
| rRNA sequences tree | GTR+I+G |
| All PKS tree | LG+I+G+F |
