# Supplementary material for: Genome-Wide Analysis of Secondary Metabolite Gene Clusters in Ophiostoma ulmi and Ophiostoma novo-ulmi Reveals a Fujikurin-Like Gene Cluster with a Putative Role in Infection
Source: Front Microbiol. 2017 Jun 13;8:1063. doi: 10.3389/fmicb.2017.01063 (PMC5468452; doi:10.3389/fmicb.2017.01063)
Supplement: Supplementary file 3 [file Table_3.DOCX]

***Supplementary Table 3***

**Genome-wide analysis of secondary metabolite gene clusters in O*phiostoma* *ulmi* and *Ophiostoma novo-ulmi* reveals a fujikurin-like gene cluster with a putative role in infection**

**Nicolau Sbaraini ^1, 2^, Fábio Carrer Andreis ^1, 2^, Claudia Elizabeth Thompson ^1, 2, 3^, Rafael Lucas Muniz Guedes ^1, 3^, Ângela Junges ^2^, Thais Campos ^2^, Charley Christian Staats ^1, 2^, Marilene Henning Vainstein ^1, 2^, Ana Tereza Ribeiro de Vasconcelos ^1, 3^, Augusto Schrank ^1, 2,*^.**

*** Correspondence:**Augusto Schrank
[aschrank@cbiot.ufrgs.br](mailto:aschrank@cbiot.ufrgs.br)

Expression profile of the predicted backbone genes.

| Backbone genes | Raw read counts | | | | | | | | | Differential expression (LogFC) | | | |
| --- | --- | --- | --- | --- | --- | --- | --- | --- | --- | --- | --- | --- | --- |
|  | Yeast (1) | Yeast (2) | Yeast (3) | Solid Mycelium (1) | Solid Mycelium (2) | Solid Mycelium (3) | Liquid Mycelium (1) | Liquid Mycelium (2) | Liquid Mycelium (3) | Y x LM | Y x SM | LM x Y | SM x Y |
| OpNRPS1 (OphioH327gp2446) | 4016 | 3181 | 3205 | 1848 | 2101 | 1385 | 3533 | 2600 | 3073 | NA | NA | NA | NA |
| OpPKS4 (OphioH327gp1880) | 339 | 536 | 137 | 783 | 1943 | 2248 | 1703 | 1028 | 860 | NA | NA | NA | NA |
| OpPKS8 (OphioH327gp7312) | 24 | 13 | 9 | 25 | 41 | 316 | 84 | 77 | 63 | NA | NA | NA | NA |
| OpOTHER1 (OphioH327gp5165) | 3661 | 2246 | 1533 | 1387 | 792 | 545 | 1986 | 1014 | 1493 | 1.2 | 1.9 | NA | NA |
| OpTERP1.1 (OphioH327gp7267) | 179 | 155 | 355 | 419 | 467 | 415 | 278 | 295 | 185 | NA | NA | NA | NA |
| OpTERP1.2 (OphioH327gp7266) | 37 | 1 | 22 | 20 | 21 | 48 | 46 | 66 | 38 | NA | NA | NA | NA |
| OpPKS10 (OphioH327gp7900) | 1 | 0 | 0 | 36 | 29 | 13 | 59 | 105 | 30 | NA | NA | 6.71 | 5.46 |
| OpPKS7 (OphioH327gp6972) | 15566 | 8683 | 9463 | 8546 | 2544 | 2254 | 16241 | 6642 | 13280 | NA | NA | NA | NA |
| OpOTHER2 (OphioH327gp5448) | 1113 | 1126 | 454 | 4280 | 4889 | 4763 | 2019 | 1479 | 514 | NA | NA | NA | NA |
| OpPKS6 (OphioH327gp6776) | 25 | 3 | 10 | 14 | 18 | 40 | 17 | 10 | 9 | NA | NA | NA | NA |
| OpTERP2 (OphioH327gp1735) | 345 | 197 | 183 | 925 | 617 | 570 | 515 | 562 | 442 | NA | NA | NA | NA |
| OpPKS5 (OphioH327gp5886) | 10 | 1 | 17 | 28 | 22 | 26 | 16 | 14 | 17 | NA | NA | NA | NA |
| OpPKS2 (OphioH327gp1396) | 508 | 156 | 244 | 166 | 343 | 372 | 380 | 444 | 335 | NA | NA | NA | NA |
| OpPKS3 (OphioH327gp1434) | 7631 | 2391 | 1771 | 1785 | 1881 | 1888 | 4548 | 4141 | 4643 | NA | NA | NA | NA |
| OpOTHER3 (OphioH327gp1586) | 476 | 822 | 622 | 679 | 657 | 487 | 960 | 795 | 595 | NA | NA | NA | NA |
| OpPKS1 (OphioH327gp1078) | 3843 | 2142 | 940 | 429 | 410 | 340 | 1532 | 1186 | 1454 | NA | NA | NA | NA |
| OpTERP3 (OphioH327gp0834) | 1409 | 1502 | 1027 | 543 | 1280 | 986 | 1317 | 1338 | 1402 | NA | NA | NA | NA |
| OpTERP4 (OphioH327gp5830) | 743 | 452 | 194 | 355 | 214 | 235 | 385 | 305 | 280 | NA | NA | NA | NA |
| OpTERP5 (OphioH327gp1753) | 384 | 272 | 504 | 440 | 488 | 481 | 546 | 444 | 353 | NA | NA | NA | NA |
| OpPKS9 (OphioH327gp8463) | 3575 | 2003 | 1505 | 2760 | 3812 | 4632 | 2604 | 3769 | 3299 | NA | NA | NA | NA |

The detailed RNA-seq experimental procedure, sequencing, data management, and statistics have been described previously (Nigg et al., 2015). Information on the expression of each gene and differentially expressed genes was extracted from supplementary material (http://www.g3journal.org/content/5/11/2487.supplemental; Supplementary Table 4 – Read counts for each gene per sample in *Ophiostoma novo-ulmi*; Supplementary Table 5 – Genes overexpressed in the yeast phase of *Ophiostoma novo-ulmi*; Supplementary Table 6 – Genes overexpressed in the mycelium phase of *Ophiostoma novo-ulmi*). Differential expression levels (relative RNA counts) between yeast and each of the two mycelial conditions were considered significantly different with a false discovery rate (FDR) at a threshold of 1%. LogFC: log of fold change between yeast and mycelium; Y: yeast phase; LM: mycelium phase grown in liquid medium; SM: mycelium phase grown in petri dishes.

Nigg, M., Laroche, J., Landry, C.R., Bernier, L., 2015. RNAseq Analysis Highlights Specific Transcriptome Signatures of Yeast and Mycelial Growth Phases in the Dutch Elm Disease Fungus Ophiostoma novo-ulmi. G3-Genes Genomes Genetics 5, 2487-2495.
