# Supplementary material for: Genome-Wide Analysis of Secondary Metabolite Gene Clusters in Ophiostoma ulmi and Ophiostoma novo-ulmi Reveals a Fujikurin-Like Gene Cluster with a Putative Role in Infection
Source: Front Microbiol. 2017 Jun 13;8:1063. doi: 10.3389/fmicb.2017.01063 (PMC5468452; doi:10.3389/fmicb.2017.01063)
Supplement: Supplementary file 6 [file Data_Sheet_3.DOCX]

***Supplementary Data Sheet 3***

**Genome-wide analysis of secondary metabolite gene clusters in O*phiostoma* *ulmi* and *Ophiostoma novo-ulmi* reveals a fujikurin-like gene cluster with a putative role in infection**

**Nicolau Sbaraini ^1, 2^, Fábio Carrer Andreis ^1, 2^, Claudia Elizabeth Thompson ^1, 2, 3^, Rafael Lucas Muniz Guedes ^1, 3^, Ângela Junges ^2^, Thais Campos ^2^, Charley Christian Staats^1, 2^, Marilene Henning Vainstein ^1, 2^, Ana Tereza Ribeiro de Vasconcelos ^1, 3^, Augusto Schrank ^1, 2,*^.**

*** Correspondence:**Augusto Schrank
[aschrank@cbiot.ufrgs.br](mailto:aschrank@cbiot.ufrgs.br)

Predicted BGCs and backbone gene sequences.

>OpNRPS1 backbone gene sequence (OphioH327gp2446)

MGEIQLSILNGEPLKLPGPELLHELLVTGAPAEDGSPSTVTQPALDYQDQIGRHVTLSYPQLHGLATVIAAEISSQLKAFNASGAGQPASSSSEDSAEPPQDKQFVVPVLIPQAPELYVSQLGILKAGGAFCPIQLDAPVERIRFILDDVKADIIITTKDMAARLPADRPIQAIIVDEMGLFDRIDAVADYVPVKIPSPEDLAYVMYTSGSTGTPKGVGVPHIAATQSLLSHDRHMPLFQRFLQFAAPTFDVSVFEIFFPFYRGSTLVCANRGEMLNDLPHVINSMNIDACELTPTVAGSLLRSRANAPNLRLLLTIGEMLTEPVIREFGAGPGRTSMLWAMYGPTEAAIHCTLEPSLDGSASVRNIGFPLDTVSAYILKIPDDEENAPPLTGDPEVAAMGEVGELAVGGYQLACGYINRPEQTAAAFIDTVKYGRLYRTGDKARITEKGLLECSGRMSGGQVKLRGQRIELGEVEQAVLRAEGCLGCAASVINGMLVAFCDVGPRPESTPDIMEELILESCRAWLPRFMIPNDIVQMADFPRLASGKVDRKRIASEYAEAHEASKSAAQSSAPAAYSYKDDLDRQLHEIAKSILGTVPDSTSVLAVSGLDSLKAIQFAAAIRSSVAPNASAVDVLESRTLAALYTRIRALIAASQEKQDESIVSHTLEAQALALDVLAVDDIQLVSECNPIQTAMLAETMSNPKAYCNWVELEFANTFARSDIVSAFEKLIQNNESLRTGFVQHEGRFVQVIRKQLSEKQIQVVSQLTRNFELDSDASLLSPFAVQVEDAGGDKINAIRVVLHIHHAIYDGWSMDLMRQHLGTLLQGGVIEQQLQYSSVVQHYREIPASRKNEAEKYWAESLNGFQPTPLPRINEWQNKNALIVSKTANMTGFGKDQIDTVAKSIGSSPQAIFQAALAWLWSSLLGSPDVVLGTVTSGRTLPLDGIESVVGPCLQTVPVRTDLSRMRTIRDLLASTHSSSRSLLAHAFLPFSDIKKTAGFRPGQPLYDVLFVYQESLYSQKSAGDIVVEVAHQDYLETKLLWEVEPVEDGFQIRSTFHTDAFPEAQVSVFIEQYSSILAHMVGNVDANLSTISSSLPQELLSARNLNYKSFDGTADLAHLVQKAAEKTPGSPALCVATSLAGDGETETLTFAELNALANRIASYLQSVGVQPGDAVAIAMEKSVNLYAGILGILKAGGAYLPLLPSTPEARAKLIVEQAKVQHCLTDSAAYSTVVSGLPLTALNLQTADLSAFSDVNPDVPVDGTRFAYVIYTSGSTGVPKGVCVPQSNICSNLDALSRIYPIPENGRFLQSCSLVFDVSVFEIFFTWIYGMCLCSARNDVLFEDIERVVRLLGATHLSMTPTVASLIDPKNTPSVEFLVAAGEPLTERVSNAWLEHLYQGYGPSELTNVCTARHMGGVLGCNIGPGLENTSSFILEVDGLDPVPIGSAGEMCFGGDQVVSGYLGQPELTAQKFIQHPSFGRIYRTGDIGRMLPDGSIMLTGRIDDQVKLRGQRIELNEINSVLRDSSLLLQVITVPVRQEDSGSDQLASFFVPSGRGLSATTTALPQLLDVSDKSLRDIVAQLFTQLSLRLPSYMVPSYLLPISGVPLMPSGKINKRSLLALFSELTKDALTATSYVSETTDADFDDVSAGQWSDLEMQLAQVLADALGADIQSIGRWTPLASFGLDSLSAIRVSKVLTRTLEKRIAISDILKNMSVAQLAQVIEKSGVKSVEPSAPKSATVFSETYLAELKASLDSHSLSAAKVLPCMPLQEAMLVSPVRGRSYVNRMLFKLQTSDLALVKEAWLTMCSRHEILRTCFSTTDHASHPIAQVVLQSYPTPWIEMLTSDADTVESLVEKHVQTLTSPVDAFQPPVSFAVIKQGDNLFLSFVCHHAVYDGEAMGLLLSEVETLIAGETLATETAPSFEPFLDQALALPESTDSFWKTHLDGFRPTLLSADKSANSENSTKPAVVFKQTLDVPLSVIQGQAKELGVSLLTTCQSAWACVLAVLSGTDDVCFGNVYNGRSIPVDGVDRLVAPCFNTLPVRAALSSLGSSKDLLAYFQTLNPELMQYQFTPLRKIQRQHLGGMRLFDSLLLLQQPSQPLDSSVWTLLKDDGEMDIPLVCELVPNQAKDEIEVTLHSESHVLPEGGSALILELFAAVSKMLLKFSSSRIPASHDLAQSLQDDLQSLNVEFERVPLPVDETATAAQDTKVVEMWTPTESKIRVVLSQLSGIPEDSIRRDTTIYRLGLDSIRAVQVASVLRKGGLQVSAVDVMEHPSCTAMASFLSKASSASNESEPMTPTSGVYTPLSSPSIASSFPPAAPVLDLTDFKQSAQPILDKHSVFIDSEIEAVLPCTPLQAGLLNEFFKSNGQHYFNYISFNKESGASTPELTSQTWKDAWRHAASFIPMLRTGFINIDSAEEASSLSPYAMIQISPVSMLENHVQVHIVKETSDTKFDLAKWRADAAHKALSNTQLPPWQVAIVESDCSLTCHLAIHHALYDAASLQGIFDTVSDFVVSGEHARKQVLTSTEAAVADILHKVTSLTGPSSAISSSWKERAPQAVVNSFPILTPLKVSAPEFQVLSRVSSQPLSDLQTAVRSAGFTLNAVLQAAWARILSSYIGDASVVFGAVLSGRNTEVTENSLFPCISTLPVIAVNETRNRKLVEQLMDTNILLHKSQHVPLSQIQRWLGQPDARLFDTLVVYQAIENQLPTAVYDSRPAHHPWTIADEKAVVDYPVSIEAIPHADNRFEYQITFDKSFLPEEHAQTLLAQFDAVVAHLAFNPDANEDDLVPVDSSLYSVIPPRDTELQSEITLMHQFVEAQASKQPNKTALQFVTAFDDNDQPISTEWSYQELNDRGNKVARLVAQYVQPGSIVAICFDKCPEAFFTMLGIFKAGCAYLALDPSAPAARKEFIMEDSGSTLLLTEAFRAQSIVADGGLLDIKPGVQVLGIDESVLKTEPSPDMAAVLAGRRETLPSDISYCLYTSGTTGTPKGCALTHENGVQCMLAFQELFNNHYDANSKWLQFASFHFDVAVVEQYWTWSVGVTLVGAPRDLILEDLAGTIARLEITHIDLTPSLGRMLNPKDVPSLCRGVFITGGEPLKQEMLDPWGPTGAVHNFYGPTEATIGVTSYPQVPQNGRASNIGRQFPNVGSFVFRPNTQTPVLRGAVGELCVSGKLVGKGYLNREELTNDRFPTLTEKSYPSYGERIYRTGDLVRLLHDGCFDFLGRADDQVKLRGQRLEIGEINHAIRLGVGSSIGDVATLVIRDEENKKDFLVSFVVVVDETAPADRRDISALKVVSGEKNSASLSRQVQTACRERLPGYMVPTYVVQLPFIPLSPNNKAEAKELKRLFNSLTPEERMRTTGSDNSSDRKAELSQSLSGKTVLDVFRKLSLVSQDATLSPDTSIFELGVDSISVLRLARAMKNAGLAAATPSLVLTHPLVGDLVAALDTQRTSSVAGPVVEARLLVDACQHRHRSAVCQILGVDSEQIEYIAPCSALQQGMISRSRSGPEHKDTYFNAFEFTLKEGVAVDQVQAAWDKVISNNSVLRTQFVATSDGFVQVVLKTNTMSWDRINLSDEDELTSVLQQRHAAWVKTNQDDIISKPLEIYSVSSEGKAIVVVHIFHALYDAASLDLILSQVAESLSGVSISAASSSEHPSFLEALTYGPLRNYSTSRQFWEDHMKGRSFKQFPVLAKDGSSSQGAVTAGRTVSFAALDQLRVSLGVTHSTLVQALWVSVLQKIYGGSITLGLVLSGRTIDDLDNVDYVVGPLFNTLPFHAARVPGQSWAALAQACYTFSTTTLPFQQVPLRDIQKWCSAGQPLFDVLFSFQFADAEQSISTKSLWTQSEPEAHADYPMALEVTLDNASDDGAPSLQLLLVAKGDVADEAALGQILDDFETALQAFSANPLQPVFGTDDIVADQTDDVATPSAVRGVAALDTSSSSFEWSNIAVSIRREMAGLASVPEDSINETTTLLELGLDSVDTIKLSARLRKAGVRLSNSQLVRGQTIVSFVNTISESIAVNATAQQANGVNKTETAEEIVAATSAALKEYLISSGRDLSSVTSILPPTPLQEAMVADMIQSNFALYFNNDILELSEDVDFELLKKSWAYTIVKNPILRTVFYTIDSPDFDISYCQAVQAVSVVDDRVITEHEVESKDAIATVANDARQRAIAGEGESNLFQLDFVHTADENRRFLVLSISHALYDGWSLDLIHTAVESTYHNGISEVSAALADSSAPAYAGQLGRIFQSSGLQADGFWKSFLNGAQPTYVQCHSPAIGSDDISEKSVVRVESASSVSADELVQFCKRQSTSIQVVGQACWAAVLSSLTSSLDVLFGVVLSGRDHEGADEMMFPTMNTVPVRVVLHGSTPDLLQYMQTNMGTIGEYQHYPLRKAQKHVPRTTESSAGESGIFNTLFILQKRVQTDANNKNEIGPLMKSVGGASDVEYPICIEMEIVSDASKGGDTIVWRTACDRSYVSAHGASRMLHQLDVALRFLIQASSTSAADGGSVLKFEDDGVSVCGLPPFSPKIAGVSSRTADTAIPVNTDSSGSWSEDELAIRSVLSSVSGLSEDAIFRTGQTLYHLGLDSISTIKVSSLLKKTKSLSLGVRDMLSASSIEEMAAIAASKKDKRALAKTTAASALSTLSSLDHAAIASAAGVSLVDVEHILPANATQTHLISVWQNTDGQVFFPEFRYRLKGKLAQGRRTIFAAWAQLVDEFPILRTTFVATTSTSVPIAQVIIKRPADRGTWSSLGSPAKSFAIISAVAEKDGQAYSLSLKIHHALYDAVSLQVLLARLQELCQPQVAQLADTYAGIPPAWVDHLSSQTSEITKQLNKTFWSAYFSNANIADPITASSPRDGRASYYRPGALPDVAHLKEAATKYGVSLQSLVFAVYAKMRASAANDDVVFGIYLANREESDNDNTALTGFPTLCLVPLIVRSPAQTNLVDIAAQVQRDINAISTPGPNGAAAPLTAALWEIREWTGISVDSFVNFLLPDVVPEVDAASYVSSSASSVTIAIDDESSVGEAATSPLVHPSMYPALQNNRVQAAYTDAVDVEVAVRDNRLDVGVFGSANKLVNDQGAQDLVVRLAEALGEEFSV

**Predicted cluster (BGC boundaries were delimited by comparative genomic analysis):** Ferricrocin backbone gene (OphioH327gp2446) and the L-ornithine N5-oxygenase gene (OphioH327gp2447).

>OpPKS1 (OphioH327gp1078)

MADLILLFGDQTNEFLPSIEELNRHALHSSNLRSFFLRSTDRLRASFARAPASYRRSLPTSFETPLELATWTSSQTSAPFFKCSPALSAALLCLAQLGHIIIELEASPRLLEHKDKHNANTVLLGTCTGLLAAIAVASSDNLADLLVIADEVVDLAFNIGLESVRRTATIDDSSASTSSWATLVTNISDITSVEAALADFNTNSVLSADRQAYISARGLTSSVITISGPPSTTSSLFSISSSAAPALAGTKLIPLGICAAFHAPHLQPVSLETVLLRSQLSSSLSGRNLRPDIALLSAGHGHQYEQSSSFTAALQQALHDIFQEPIALETAVSSLSKTITAPAVTLASFGPANSTKAIARHLESNGTTVNCKANSTSSSTTSDHTASSSPSPAPPGDNAIAIVGMASRLPGSETLDEFWQVLEKGLDLHEPIRPDRFDVDSHCDITGKRRNTSLSPYGVFIDRPGYFDTRLFNMSPREAAQTDPQQRLLLLTTYEALEMAGYAPDRTPSTAARRIGSFVGQTSDDYRDVNASQDVDTYFITGGIRAFGPGRLNYHFGWEGPSYSVDTACSSSAASIQLACTSLLSRECDTAVAGGANFLTASDLFAGLSRGSFLSKTGGCKTFDAEADGYVRADGVAVIVLKRLSDAIADNDNILAVVRSAVTNHSAEAVSITHPHAATQERLFRAALNRAGLHPHDIDYAELHGTGTQAGDATESRSVTNVLSPGRGPAQPLYVGTVKPNLGHGEAASGVTSLMKVILMLRNNAIPPHIGIKGNMNPKLPPFAELNTHIAFKKTPFLPRSGGDGKRRILINNFDAAGGNTSIIMEDAPARRVVAIAPSDPRSHHIISVSGRTANALIGNNQRLLDFVRRTPDIKLADLSYTTTARRMHQGLRQTHVVSTLDGLVTSLEAAARDEERTKKARAPQQPPSVVFCFTGQGSQYAGMASELFNSQPTFRETLLECDRVSTSQGFESFLTLLESDSLESALVGASPVQVQLAIVSIELALATLWKSIGVTPAAVIGHSLGEYAALCTAGVLSLSDCLYLVGRRASLMLSSCTPGTQSMLALASSLAESEALLSSNPAFSSCEIACANGPSSTVVSGPAADIAELQKATSVKTTLLDVQFAFHSAQMDPLIAEFSQYTAKAHYASPSIPFASTLLGTIVDSSEDTPTINGSYLTRQMRERVRFTDALESLLASKSIQASQSVWIETGPSPICIGLARSTLRNNAAISSPLLLPSLKRGDSDWKIFSQAVAKVHEAGVKVDWQEYHSAFESSLRLLELPTYAFDLKNYWIQYRGDWALRKGDLAIEPESIAPEPKVVSYPAFVATTGLHRIESQVISNNGSIAVTFATDAIEPKLNKALRGHLVNGAGLCPSSVLGDMALTASNYIRRLSHLLSEKSGSVSPLNNDLCLDVHEMGIHKPLLIQPGQTKQTILTTAVWESPSSSVTFTFSSRDGPDGHKEDHAHCKATFSDGDAWKSLWKRSSYLVQGRMDQLVEAAATGRAHKLLRPMVYKLFASFVDYAEKYQGLQEVYMDSHQLEAAANVKFLTNDSDGTFTCSPYWIDGFAHLSGFILNGADTTPADSVFISHGWGSMKFTRQFSADKSYRSYVRMQEEASSKGVFSGDVFFFEGDEVIAMCEDLKFQRVKRSILAYLLPSGAAPSRSTAPITPPASPPRKAAAFKAGSPVKRKAAPKKAVISQESLLFSDILNTVTSEVGIEVGELADDAWFADLGVDSLLAISITAKLSTLMGRHLPATLFTECLSVSQLRTYFADEVGTSSTGGSNSAAYDTPPQSEPGSDDEESDTVPSSTVSRGDTPFSSIGQTPEPGAASDHSSELFRKIIAQEVGVDASEIEDDTHLADLGVDSLLSLSILGTIKAQTGRVLPSSFLIDHPTLNDIQKVLGGHSHTSPQALAQAVEKAATGAVPEKKMAAAKHAAESILLQGSPSSSSPALFLLPDGSGSASSYVGLPPLSLKGAVYGLNSPFLKHPEDFTVSLQDVASMYVDEIRRVQPSGPYHLGGWSIGGSYAFEVASQLSARHGGQINSLILIDAPCPKSLPPLPTQTIDLLDKIGAFDGLKGRASNKMRSGVRDHFAGSVNSLKQYRPVPIPSQAIPKTVSVLWARDGVWETVGDDVRRQNGGIQGGINAAEDWIMDPRKDKGPNGWESLLPGARIECKIVPGDHFTIMRRPGVTALGEQVRCCVSE

**Predicted cluster (antiSMASH prediction):**

| OphioH327gp1074 |
| --- |
| OphioH327gp1075 |
| OphioH327gp1076 |
| OphioH327gp1077 |
| OphioH327gp1078 |
| OphioH327gp1079 |
| OphioH327gp1080 |
| OphioH327gp1081 |
| OphioH327gp1082 |
| OphioH327gp1083 |

>OpPKS2 (OphioH327gp1396)

MGDRLAFLLFGDQSLDTYGFLSGFYRQTDQGILAKAFLSQTGEALRKEIETLGRLERSKLPVFKTLQQLNERYHSQSLKHPGLDGALLCVTQLAHYIDHAEKNWEDVTKHDHTFLVGLCSGMFAAAAIASTPSLSALVPVATQAVLMAFRTGLHVSKLADQLCPPGKASGSWTYILPNQTEDNVAHVLSDFNKSNGIPAASSVYISAVSSSNVAVSGPPSTLKALTAAGVLGRSASIPVHGPYHAAHLHRAADVKKILRLSDPAVADVFYKTKPRSAVMSCATGDWLSEETTEGLFEAAVYEILNEPLCFGKVLDGCLDLATGYISGDCLVIPFGPTQNANTLANLLRSKIDITVSLRPSPDYTSSKVNIGSSPSKIGNHGSSGKCKLAIVGMSGRFPDAASHEKLWELLSKGLDVHRQVPADRFPHATHVDPTGKAVNTSHTPYGCWIENPGLFDPRFFNMSPREALQTDPMQRMAISTAFEALEMSGYVPNRTPSTRLDRIGTFYGQTSDDWREINAAQSVDTYYITGGVRAFGPGRINYHFGFSGPSLNIDTACSSSAAAMNVACTSLWARDCDTAIVGGLSCMTNSDIFAGLSRGQFLSKTGPCATFDNDADGYCRGDGCASVIVKRLEDAEADGDNVLAIILGTATNHSADAISITHPHGPTQSILSSAILDETGVDPLDVDYVEMHGTGTQAGDGTEMISVTDVFAPADRKRPADRPLYLGSIKANVGHGEAASGVTALVKVLLMLQKNAIPPHVGIKKEINKTFPKDLSDRNVNIAFHMTPFRRTDGKPRRVFINNFSAAGGNTGLLLEDAPRRTAPTADPRGTHVITVTAKSKSAMIKNGERLLAWMAENPETPVADVAYTTTARRIQHNWRMNITATDLKEAQGALQQRLKDNFNPVSPEQPKVAFMFTGQGSHYASLGKDFYAHFSVFRTSIDEFDQLAKVHGFQSFMPLIDGSEPEVSKLSPVIVQLGLACFEMALARLWISWGIKPAAVLGHSLGEYAALNIAGVLSASDTIYLVGSRACLLVEKCTAGTHGMLAVQGTVATVTDALGARASSVNVACINGPRETVLSGEAAQMAEVAQELGAAGFKCTQIKVPFAFHSDQVEPILDDFEKLASAVSFNAPTLPIISPLLGKLIPANGSASIDASYLRNHAREAVNFLGGLVSAQQSSAIDEKTLWLEVGPHPVCANMVKAAFGATTIAVPTVRRNEAAYKTLSNSLCTLHSAGLNVDWNEMHKDFAASTRMANIPTYAFDEKNYWLQYEGDWSLIKNRFAQMLPAAIGGPAGSAKPTLSTTSVQKVTHEEVNGSTVTLDIESDLCRADLRLTLEGHLVNNTPLCPSTLYADMAMTIGDYVYKLVHPEAEKVYTNISEVEVGKTLIFEDAAASQILRVSTIAQIDKGYADLVFHTGEGKKRVEHAKCKVYYGNAAQWSNEFERVNYLIRSRIDALKEGEKRGTASKVGRGIAYKLFTALVDYAPRYRGMEEVILDSATCEATATIRFQTSEKDGNFFFNPYWIDSMCHISGFIVNATDAVDSREQIYISHGWGSLRFIEKPDPSKTYTNYIRMQPVKGTTMYSGDAYVFDGDRIIGVCGDVRFAAIPRKVLNLVLPPRGSAGAVAPAPVPAARKAAPVAAAASKAKSKNTKTVTHANIGKVNQKLTSVVIQVMDILATEVGVSHDELVDNVAFGDLGVDSLMSLTVSGRIREDLEIDLHSNAFVDHPTIGAFKKYLAQFEKAGASDSLEDSGNSSSDSETDGMKSDSGVTTPMEESDTGSIKDGGEVSLDGLTAHQLAVRNTIAEEMQVDVDEIIAAPDLADLGMDSLMSLQILGTLREKTGLNIPSDLFVSNPSLKDIERCLGIEPRPKRIVAPTPAPVAPAPAASSSQVIRLEPADPIEKLPPPTTYVDHYPHRKATSVLLQGSPRSATYNLFMIPDGSGSATSYTEISEIGSKWAVWGLFSPFMKTPEEYKCGVYGMATKFIEEMKRRQPEGPYNISGWSAGGVIAFEIINQLTKAGDKVDNLTALSNYTAVPIPAEKCPKITIIWCEDGVCHLPTDPRPEPYPEGHALFLLDNRDDFGPNRWDEYLDAKKMTIWHMPGNHFSMMHGEYARQLGALMREGLGL

**Predicted cluster (antiSMASH prediction):**

| OphioH327gp1395 |
| --- |
| OphioH327gp1396 |
| OphioH327gp1397 |
| OphioH327gp1398 |
| OphioH327gp1399 |

>OpPKS3 (OphioH327gp1434)

MADPVQPVAIVGLGGRFPGKATDPLKLWEMCSNGEDAWSEVPSSRFNHKAFYHPDQSRSGASLDPQQRLLLECSYEALENAGMSLEDVNGSDMGVFVGSFCFDWAKVTLRDADAIPLYHATGTGQAMLANRLSYFFNLHGPSVTLDTACSSSLVALHQACQALRAGECDTALVAGVNCTLCQDSLASMSSMGFLSAEGRSYTYDSRAAGYGRGEGVAAVVLKRLDLALDSGDAVRAIVRNTGVNQDGRTPGITFPSGEAQAALIRRVYDQAGLDLAATSYVEAHGTGTQAGDPIEARALYNTFGASRPNDGTIPPLVVGCIKTNIGHLEGASGAAGLIKTVLMLERETILPNCDFREANSKIPLHEWRLTVPTELQSWRAASGGAAVLRASVNGFGYGGTNAHAIVESAADYLRERGLDGGKYKFHHGHTYKTAQITTTSNDTATDSGSSSNDDTVHTPPPSIPSTPHSEKVVPEPLSGPVAPVPSCSTSVDDAPSTATPAPAATDDNSSVTEPNPSIDTSLTVPKLFVFSAQDETTGKAQIAQLQSYLAQDSHHDSLPHNAAFLNDLAHTLNSRRSALPWKAAVAATSAESLQAALAGARFQNASRPPSVGFVFTGQGAQWYAMGRELATTNAVFRASLDRSAAILASSTMKAEWSLHDELLRRDAETTRVNEPAVSQSLCTVLQVALVDMLRSWGIKPAAVVGHSSGEIGAAYAAGLLSQEAAVAAAYFRVPLDSSAADSCRGGMMALACDEETTVRLLDSLETGKASIACYNSPLSFTVSGDDSAIDELVVKCKEQGIRSRKLVVGFAYHSHKMIGAATGYRELLLANRTIVEAFSNPTAEAGSIEMYSSVTGKRLAPGALTLDYWVDNLVNPVRFTEALTALCYEAQTGESIDDPNFTVRKPQVDVLVEVGPHAALAGPIRQILQSQTPLTKIGVLGMLRRGQDATETSQLLACQLIEKGLPLDIAAHNASYRTEDSDASLEIAPVPLVDLPPYPWNHATAYWAEPVESIRYRQREHARHDLLGAPVRFGSPLEPRWRQWIRTAETPWVRDHRVQGLVVYPAAGYISMAIEAARQDALHKAGPVGSKEAVAAAIQIDGYELRDVTLGQALIVPEDSGEVEAMISLRPLSESAMDNSTVWHEFFVYSCSTSATNTTPMGAPITDNWSEHCRGRIAVRWKPRSDVAETSLVTDTAAIRAETARRDAANRTVIINACQTDVELESLYDHCTAIGLEYGPTFANLVAAKHAPSTSKADPINRILGTVTIPDVAATMPAHHHSPIVVHPGTLDACLHGIMSFRDLLKAAIMPVFFENIYISAAMENVASGENIGVYLGVQRSGFRNLQIDLTAYGPDAHKPELDACSESLAAPLIRMEGLRMTSLAGSMPSGSDGNLPPKSYFESLWKPDPVFLSSEHFNKLCAHLIPTEAESAGLRRLEQSAYYMSDYAISQVPKEALPTLTNKGGKLYKSLMRLRNSVLALHEERISERAAAGLTELEPARADDIPTWPNATPAERAVLLSELEATGAEGRLLAAVSASMHRIILGTADPLEVTMKNDVLGKYYAENPRMLRQYQQAAVYVDLLAHKNPHMHILEIGAGTGGATIPLLRALGGDADGKNTSPRFASYDVTDISSGFFEAFREKVQAWQNLINFKRLDVEKDPSRQGFTPGTYDLVIAANVLHATRNIGNTVANARQLLRPGGTLMLIELVRVPEQKVATAAVGNMFGIFDGWWIAEEENRQDSPLLAETQWDSVLRENGFSGLDAAVWETPDLATHQGTTMISTAIYQPSSITEKATSSTETIVEVEETETVSEPTTVVAVPSGIPPTVLVTDSVANKSWLQSLAKALFVINPSAVEFPIYTIGDKIPSFDAETVCIFYQTEASTVGIQTPETMENLRRLFIRSTEGSGGSVLWLTTGATDGDNAPDFSLVQGLLRTLRVELGGRLIHLDLDMEVSADNSTVYESIVRVFERSFAAKNLPDVDNEDHRAPNTEPELELAIRGTNSTVYIPRYDENLVVGNYVASRTGRRNLVPASPVQPGRHLKLEIGQPGLLDSLFFDDDKNAIGNPPLGPDQVEISVRAAGLNFHDVMVAMGQIETRDLGRECAGVITQIGSLVTGLKVGDRVAAPADGTIATTVRCDAWRAQRLPANMSFEAGASLPIIFCTALHTMNLSNLTEGETVLIHAASGGLGQALIQLCQERGAIIYATVGTAEKKQFLVDQYGLEPSHIFSSRDDSFASAVMQATNNEGVDVVFNVLASELLRASWRCVASFGRFVELGKRDLTRNGRLDMGQFTRNVTFVAVDLVALMAEKPKYSAKLWAQCMDLIRKGVARAPTPLTSYGYADAHLAMHTMQTGRHIGKLILVPESQPITKTTAKRTLAVTEGEDGTSVTTTTVTKTEVAPGPYSLATKVKIAQVEPVKLRADASYLLVGGLGGIGRALSARMVKELGARYLIILTRGGVSNEVAAASVADLRAQGASVLVCNCDVGQAEQLAAALKTAEENGFPAVRGAVNLSLVMESALFQDMSWQSWNNSLWPKVPGSWNLHNALSGNEIDFFILVSSMVGTIGNPSQTAYGSASSFQDAFAKWRRRRGLAGVSIDLGMIADIGYVAEHGSVQQTLLSQGFEEIDGPECLAILEAAMLAPTEFDPKWKAANVVTGLGLGRYSGGDPSRAVYTDPRFRLCRRMATTSGGDEDSPAAGGEETQSIREMLRQAASLTDVISTLETAIRSKITVLLMLPAEDELDTQKPLSQYGLDSLIAVELRNWVNSEMEATVPVLEFLGSRNIQSLSGFIARQSRLVRKELLAE

**Predicted cluster (antiSMASH prediction):**

| OphioH327gp1429 |
| --- |
| OphioH327gp1430 |
| OphioH327gp1431 |
| OphioH327gp1432 |
| OphioH327gp1433 |
| OphioH327gp1434 |
| OphioH327gp1435 |
| OphioH327gp1436 |
| OphioH327gp1437 |

>OpPKS4 (OphioH327gp1880)

MAGTKAIPEPIAVVGSSCRLPGGSSSPSKLWTLLKEPRDLVREVPHSRFNVQGFYHEDGEHHGATNATHSYFLDDDEDVRAFDYNFFNVNPREAEAMDPQQRILLETVYEGMESSGYSIQSLKGSDTCIFVGQASDDYSNLLNRDLDTIPNYFATGAARSIMSNRVSYFYDWKGTSVCLDTACSSSLVAVHLGIQELRNGTSKMAVAAGVNLILGPEVYIFESKLHMLSPTGRSRMWDAGADGYSRGEGCVAVFLKTLSQAIADGDHIECIIRETGLNQDGHTPGITMPSPDAQAALIRATYARAGLDLSRKEDRCQYFEAHGTGTPAGDPVEAEAVYRAFFSDAEPQTDADEPLYVGSIKTVLGHLESGAGLAGFLKASLAVQHGIIPPNMHFNSLNAKITPFYHRLNVPTKALPWPQLPKGTPRRASLNNFGFGGTNAHAIIEAWEPETARADVVHVTETTNSNIPYGPLLFSANSKSALVASTKAFSKYLSDLDEDAPLSLSDLAWTLQQRTAFSYRAYVSGTTRAELIDSIAAGLKIASRPEEAGPNTFVTNALLVTKALPARILGVFTGQGAQWPAMGSALYAHSALFRSSLQSLETSLAELPEADRPAWSLAEELIKPAATSRIKEAALSQPLCTALQIALVDLLKAAGIEFSGVVGHSSGEIAAAYAAGYLSASDSIRIAYLRGIHAEKAKTGPKPGKMMAVGMSYDEAVTFSNQDKYAGRIVAAASNSRSSTTLSGDADAISEAEAQLKADGVFARVLLVDTAYHSHHMKLCSDSYLESLKQCNITVLAPDVAPDAGFTWFSSVHGTDGRSIYEPSTFKDTYWVDNMAQPVLFSQALDRAITESHCFDMVLEVGPHPALKGPASEVLRTLTGVDLPYTGVLSRGANDLSAFSSALGFVWSHFQSSEKTVVDFDGFREACVGSSAALQTSETPKTKPRLVKDLPAYVWDHDVPLWKESIVSRKFRLRDDRPHELLGTVTPVGNNQEMRWRNVMKINELEWLRGHVFQNQILFPAAGYVSMAVEASVHLAKKNTTTMSGEVAETVQIVELQDLQIHAAITIDADAAGTEVLFVIRVVDRDVSLHQIIAEFNCYSGSVDGTTNDAEKVNFSGRAVIILDSVEDASVLPPRVAPTLPMANVDVTRFYRELEKIGLRYSGDFVVESAQRRLGSSTAMINRPSTNSQLIVHPATLDAAFQGVMAAFCFPGDGRMWTSYLPTGIRTVRVNVAAAKLRMQESGENVSQAVADCFLRDATSKVISGDLDLFNANDSSLCEVQITGLTCSSFTKPGPKNDRKTFSKMVWRPEVGSGISPEDQPLVKNEMFEEINILDRISVYFLRRLLEEVSEEEFAAAEWQFQSVRGWAKDYLLPKVDSGQHRRLKPSCINDTAEMVEGWVEKYGHTVDMELAWAVGKNLPAMVRGQVPTLQVLMENNRLNRLYKDGVGIDLVYAQFNALVKQMNHQHSGLRVLEIGAGTGGASVGAIKALSPHFSSYTYTDISPAFFEKSKQQFSHEELAKMEFRVLDIERDPLEQGFDEEGYDLVLASNVLHATEFLSRTLANCRKITKPGGRMIIIELTGDAIYTSFIMSGLPGWWLGRNDGRRYGPLASEARWDMLMQEAGFSGVDVSCSDLDDFYGLVMSTQAVDNRISLLRDPLSVGTWDNAGSTVAASPITNFVLVGGKTISVNRTAQTVRRLLRPLGVDVLLVPGVEDLETQEIPAGAAVLCLSDLDEPMFKDLNEPKFKGMQNMILNSSALLWVTKNRRTSEPYSNMAIGMARVIAFESPHLVMQFLDVDDFQGGLGNKATAEATLFAETLLRLVYLNSPDFSDVFWVRETELCLQDGRVFVPRIVDDDDLNDRLNADRRAITKKLLLGTSNIEVVQESGSIHVLESEPIHLSTSVRTTEDEEASHQIKIQASSLYPVRTRDGKTFYVGVGQDLGADNKAVLVLSADNGSVVQVAPANIIEIGGDPEVTAAGKPELLQDVLRDLVAESLTSDTEGTLWVHGGDSDLVERLVTKTAEKSIKLVSTTASGISTEARHRKQVSIHRYDTERRVLSLLPKGHLTYANLNVAAESEALSRIVRNTSSLSSETTVDVRHLFKTVQNCQSVGLSLSLLELTAALRRGLKSAKIAKDNVASLADGIVGIENVPNLPVGSLPPTQVISWTTSTPVAAEVFPTSSLSLFKSDKTYFLVGLTAELGLSICGWMADHGARHIAIASRNPKVDEAVLEDLKRRGASDLRVFALDVSDKQSLLDVHATIKAEMPPIAGVANAAMVLRDKPFDNCTVDDFKYVLGPKVDGSRYMDELFSTPGELDFFIFFSSLASVIGNKGQSNYAAANMYMHTLATQRRQRGLPASVIDIAMLLGVGYVDRALDHLESRLVGVYGYVGLYEPEMHNIFASAILAGHPESSIGDHAPQIMTGIPINAGRRFPTKPLFSQVLQEEEQVSTAADSKTELSARVLSQLAEAANKGDATLNILEAAFSRKVELILQLPADKIETKVPLIKLGIDSLVAVELRSWFLKELNIDMAVLKFLGGSSILDICRDALGQLSLSSAPKDVKQAVEIKEVEKTTTVDTKDTTAAPIVASKAITEPIAESTPKVAVAATPEPLSDSTTESVAGPATESSSEPAERVIDATPEVVSEPAFEAVVPVEAAPAVEFVESSTEVAEPTIEVAEPTVEVAEPIIETPETLEIDVEATEPTIEATEPIVELTEPIVETPEVVVEAPEATAEVSTLDTEILVEPSVEAPAEVAVETAPAPTDEPAIESVAEPATEIAAEATPTLAEESAADATNTTGTELETETAIKHEITKLIIEPEATAALTAEPSIGTSGTTSEDTSYSPTTAPTESSSDDGRPRTPLAFGPLHTINTIIDTIVDTHEHAGFCQRGQISLSLKENERSTKMMKL

**Predicted cluster (antiSMASH prediction):**

| OphioH327gp1873 |
| --- |
| OphioH327gp1874 |
| OphioH327gp1875 |
| OphioH327gp1876 |
| OphioH327gp1877 |
| OphioH327gp1878 |
| OphioH327gp1879 |
| OphioH327gp1880 |
| OphioH327gp1881 |
| OphioH327gp1882 |

>OpPKS5 (OphioH327gp5886)

MPYKPAPSEPIAVVASSCRFTGGTTSPSKLWDLLKEPTDLTRPVPPDRFNIKAFYHPEGDYHGTTDAPKSYFLDDEQDPRAFDTGFFGIAPKEAEAVDPQQRLLLETVYEALEASGYKLPEWTGKDVAVYVGAMTSDFDSMSQRDDLTTSPYYATGNARSILSNRISYFFDFHGPSATIDTACSSSLVALHQAVLSLRAGDCSAACVAGVNLMLAPEQFVVESSLHMLSPAGHCHMWDTRADGYARGEGAAVLLLKPLSRALADGDASRIQGIIRETGVGSDGRTSGITMPSPDAQASLIRSTYQRAGLDIADPADRCQYFEAHGTGTPVGDPREAQAIHSAFFGPAASNIPEDQEKLLVGSVKTVIGHTEGAAGVAGILKVMLAMKNGQVPPNLHMLQLNPTVAPFCTPSPTHNGHLEVVSKLQAWPQPSPGQPMRASVNSFGFGGTNAHAIMEHYDAYIHNPEWSLLSSPSTAGEFGVRSRRIDKPLKLLGIFTGQGAQWAGMSKHLLLTSGIYREAIQSLDKILRSCPHPPTWSIETELTKLESDSRVKEAAISQPLCTALQVGLVTLLQHLGIRFACVVGHSSGEIGAAFAAGRISAKDAILIAYYRGLSVSDLDMQSLKGGMLAVGLSYADAIEFCQQFDGRIGVAASNSPSSTTLSGDIADIEVAEDQLKSDKIFARRLVVDKAYHSHHMANFSEIYNAYLAACQISPLYSSSEEEAERPAWVSSVYPDGDAPTNDQLASRYWVDNMVKPVLFREAIDRALSLEGLEDGIDGAIEVGPHSTLKGPFLDTYAARAPPPTSTAKNTPVYTSLVRRGQQDDIVLQNFIGFMTTHFDPSPVKIEGLLMPDEQDYLNISLAKMHSNDAEKLPGYVWDHSQIYYRQSRIVRQFHNQTEAPHELLGTRTRDDVQGCELRWRNLLRVDKLPWLAHHAFQGQPLFPASAYCIMALDAAKALLAGRAASLVEIEDIEFVGGTPLEIDGPATEIMFSLMVQDPSKMAAPTTTNSEETIKAFFSIYATPEASDVPMRKRCSGCIRVVLGEPDKDAVPCRIDPSEQPETFPVPIDSFYDMMQKVGLSYSGPFEAIESLQRRHDFASASLKRFHPEDTTGLAISPATLDSCLQACFATFSSPGDKALWTAFLPIHIKKVTFNMAHKGVASAAGPKRTSMSASSVDGGNYHVDAELTHFQEAALDSPSRITGDILIYNDDGDMEVGVEALTVGSFASSDPSTDRELYLHTTYAADPEHSLVIGPATSHMEEDGAVDGFHDANYRMLEESCERVAAFGRPNSAWPAETKETLEQYILTSPYSLTLQKIHNMAPVDVMDQQYQTILEEGRHAGQFQAQLSKIVKQIGHRYPRMSVFSLGDVEMCLSLPIVCGLGSAFTRYTSATVGTDLGLAKHLKKQPEYLQQKVHSISLDAAQEIKETLSSGTIITENAPSDLVIVSTSIFKQLESSEAVLSQIMKLMRPGGFLLLVHMPLTTATNRKNTAKFSAVTDDEQLSVSSNGDAMITPPDWPDLLEQSGFMNPDIDNTDQHYPGGFSITVRQCDSPFKKSLAQASQSDIKLDEKLLIIGGATAGTVSLASAASQILSNRLAHDSTVVYGLDSLAEMSPATLASFTSALVLADLDDYQPVLSTLDEQRLDDLRSLLLVPDLTMLWITKGARNGNAECAASYGFTRSIRAEMPSLTLQMLDFDHLHMVNGQLRAPAPVKISGALTSSATALHPLAASVIADQFIQLILAKAERDHARKDSKENPSHLWGLETELFINQDGQRLIPRMVPFAPGNDNFNASRRVVTRQVNTLSMCLEVISDPSTTYSTRIGPSFTSLIESSHLDLDLDVINTFYSTTNPLYNGFYLCLGRSELSGHVVATMDDSNASLVSVPRSQTVPLGNSTLLFDKNPGAFASYLAKYLLCQAISQHIDRSKSLVLIAPDDLLLQCASSLVTDSQHIIASIQVSARKTKHTARNTNMINIHPRSTASQINSVLRRFGEGTAFVNFLPADHFISRYIEQNLADNCTYQSWSQIGKSHKTTDDNKSSRGNREEAYGVLKMAVASASQHVGHISSASTVLEVTSLPALLMGTDNGASSPLIIDWRANRLVPHTEQPLEDFGGRRTTAVASEIELRAKRTYVLVGITRDMGQSLATLFVRQGARHIVLASRTAVIKQPQWAKKLSIDYGAVVRFMPLDVTDLAAVRRFRATIEQDLPLVGGIVNGAMVLDDRVFTHMTADTFARVMRPKTVGSRNLDTVFRDDGVDVDSKLDFFIMTSSFAAPGGHAGQSNYAAANMYMNGLAANRQRRGVAGSVLNIGVIYGLGFLHREKGELYAGLEREGYPPISERDLHHMFLEAICQGRPKTLRADRARPIIDLTTGLSRYNPDQSADNALHWHNDPRFSHFTVRSTSTADTAVDYKGGDNSAIKILTDMISQATATADVIGQKIVAALIERLSTLLHLDSGSVIRGDNSLAELGVDSLVAVETRTWLYRTTGQDVPVMRILGSPSINKCKCYHLDRTIQFYHRWLTVQCAWTLVMQSSLAAEKQSSTDRVKGLF

**Predicted cluster (BGC boundaries were delimited by CASSIS):**

| OphioH327gp5883 |
| --- |
| OphioH327gp5884 |
| OphioH327gp5885 |
| OphioH327gp5886 |
| OphioH327gp5887 |
| OphioH327gp5888 |
| OphioH327gp5889 |

>OpPKS6 (OphioH327gp6776)

MGTEGEPADGCIDDAIAVVGMACRFPGDATNVESFWEMIRDGRDAWSEIPEDRFNAKGWYHPDPNRPGSFHIKGAHFIKSDIAAFDAPFFSISTAEAVSMDPQQRMLLEVVYEALESAGIPSSSLSNTATSVFCGSFVRDYEQPCMRDPDTTPPYSATGNGIAILANRISHAFDFAGTSQTIDTGCSASMIAVHQACKSLLSGESNVGIATGVGLIFSPNTLVPMANLNILGTDGRCYTFDERASGYGRGEGTGVIVLKRLKDAVAANDTIRAVIRATASNQDGHTQGITLPSKERQVENMTEVYRGSGLDVTRTAYMECHGTGTQVGDMKETQAITGLFCQHRTPDNPLMIGSVKTNIGHLEGSAGVAGIIKGILVVERGFIPKHLNFNKPNPNIDFEGMRLKIAQELTPWPVPGLRRASVNSFGFGGTNAHVVLDDAPHFLSEAQITGANHNTFLFPGDRLKLNDLYNFHGPLNHLFVFSGHDEAALTRVIESQAGYVAKQTQQPAFETNYTYTLLDRRSRLNWRTFVVASSTADLLNKLESKTTVQPVRGPQRVPSRVGLIFCGQGAQWYAMGLELLSYHTFYVSISSASWYLKSVLGSPFSLWDELRRSKEQSKIDDPSIAQPATTAIQVALVDLLLSAGLEPSSVVGHSSGEIAAAYAVGAISRENAWKIAYFRGQCVSRIETLAPCLHGGMLAVALSEEDARRGIADAGVSLVDISCINGPLSVTLSGDACGITSMQEYFRKKGCRATRVNVDTAYHSHHMAIIEDLYMDCLKGMEPCSPKTNAVFYSSVFGRPASACELDAEYWVKNLTCPVQFFSAVKSMMKLSNPDLFIEVSPHRVWESTVHQIHSALDRPDATSYISLLHRNKDASETFLEAIGDLWTKGLQINLDWANSPCGKRPRHLVDVPSYPWDHSKKYWHESHLSKTNRFRKHGREDIIGAPLENATPQDPRWRGFFRVQENPWLEDHVVQMSVLYPAGGLLAMAIEAAKQMADPKREVLGYEVTNFKIIKPMLIPSTIQGLENMLSARIVDDDDEGDRTHGSNTAVYNFSIMSKPTDGPWTTHAKGRFMTVYCKSGETPDASMEAMHGTQAHREAFRLGQARCHTKYNPRQFYEMLDIIGMTYGPQFRNLVQIEKGDTSAHTVVKIPDTRSRMPFQFEFDHIIHPATLDAMIQSVLALGDDGEAMLPCAADRVFVSANLPKGAGARFRGYTTAEKNAARGATANITMFDDELHAPAVIIEKLQLKSVSGRAGSGSDSFLPAHRNLCSEIVWKQDSSSIAALDAAFPTTILGLVDAAAHKNPALSILFHISDTVIPSFDGTKAKEAELHQLEIVAETLKATNIVFDNLTAGYCTPRFSQCTLSGVSAKKVWQHMRLLKGTHPGFQRMQYSAVVAEDAKHDLVLCSIVDNVTMESLSMIFDKVADNGWLILLSKLGEAIGCTQYEAIRYSLHAAGFKVDGMPAKSCVFISAQKVKPYSNDVNAVNAIDGHSPSLHNVIFLVPNSPSDAVNALKKALRPRIEDQLSVKVRDVSACSLATLEEQDRNTLVVSFVEIDFALVFSMDQAIYNGLHRLLNRSRGVLWLMQGAQVGVMCPERAPFTGLARTLRSESNKKRILTLDIVDNLSLTSAKAPNLSFPSSSGGLVRAINHLFKHLLDSKSPSELGDDVEFAYADGHLLIPRLMPMTLLNHAIENGTSRSSINNKVPLLSHGRALELDASSLGSQDGPYFMEDTVSLRRPLCPTELRIAVSGTNLLPDDILAASNGISVATIGTDVFGFVLEAGSECSQFKPGDYVSARMRDTLRTHVIVDVKLVQRIQGSDKWNAVCPTALATVIYAMHTSRKIKDGEHVLVFAPMTAYGQAATWMVRTLGGHVLVACSSAAERAVLKEHFKVPVKSIMDALDGPDAQGSDRKLQDQVMSLTDGKGANIVFDPTSRHLEQAFSCVAECGRVIRVAQRDSKNPNQSHFCLPSKNASLETVDLDLLDARRSDEALRLSSDMDKLTQMPSKLHTVSHFLEYNMCDVQTAVQHATSNLYSGAHVLYTAADVGQNMVNTSVQLTQPTELSWAWTYVLVGGLGGLGRSIAEHLVQKGARHIAFFSRSGACTPEAQALMAKLREKGVQVAAFATDICNEKQLAKTVSEVERFMPPIRGTIQCAAVVDDTSFPSMDYDRWRRAFEPKTRGSHNLHKLLPKNMDFFIFLSSSSGVIGNRGQANYSAGNSYQDALARHRSLHGMHSVSLDLGLVLGAGMVAENESLLDAMKASGFIGIRITDVLFLLDRAMARPETEQKLKLPPQIVTTWHGARAWFLMILIRMSRWAATALTR

**Predicted cluster (antiSMASH prediction):**

| OphioH327gp6772 |
| --- |
| OphioH327gp6773 |
| OphioH327gp6774 |
| OphioH327gp6775 |
| OphioH327gp6776 |
| OphioH327gp6777 |
| OphioH327gp6778 |
| OphioH327gp6779 |
| OphioH327gp6780 |

>OpPKS7 (OphioH327gp6972)

MAPIAMTPDTSASPSGESDNGYFDSDNGRYVNDRASTNPSRKSSVDTDAQGAAANDAPKAEKPMPIAIVGIACRMPGDVSSPAEFWELLSRSRTGFSEVPQKRFNADTFYHPNAGKGGSFHAKGGNFLSCDLESFDAPFFGLTEKEAISMDPQQRLLLECSFEALENAGIPKQNIVGKDVGVFVGGSFPEYESHLFRDSDTIPMHQATGCAYSMQSNRLSHFFDLRGPSFTSDTACSSSMVAVHQACQSIRLGESSMALVGSCHLNMLPEFWVSFSSCRLLSDSGRSIAFDERGTGFGRGEGCGMVVLKPLDQALRDNDPVRAVIVGTGLNQDGKTPGITMPNGEAQEALMRQVYRQSGIDPKDCGFVEAHGTGTKVGDPIEATALHNVLGQGRSVRDPLYIGSVKSNIGHLEAASGIAGIIKAALMLERGFILPNHDFKIPNPKIPWKEWNLKVPINQRPWPRGKKYISVNNFGFGGTNGHVVLQAAPFRAKPSFERDLSVPMSKQRKLYPFTANDKATLTAVMKNAVIYLEQRPEIFQADLMDNIAYTLGQRRSHLQVRSALSSTDSFGLIEAINGEKYSSNKELEPLRIGFVFTGQGAQWWGMGRELFQQYPVFSSAIEHADRCLRILGASWSLAEELSRDAETSKVNEAHISQPACTAVQLALTDLLASWNILPDAVAGHSSGEIGAAYAAGIITFESAMAIAYHRGRLIPVLKAEHPDLKGRMMAIGGSKEEFQPIIDSLKEKEVRIACYNSPSSLTISGDEPALAELEKICEEKGAFNRRLMIDTAYHSHHMNLVAKEYQASLQKLKAPADTLVRFHSSLHGRLADPSELQASYWVDNLTCAVRFDEALQSMLEPIGEYRTGVNMLVELGPHSGMQGPIKQILKHVGGPAAKVPYASALIRKKDAIETAMDVAAAVFTRGLAVDFEAINFPRAHSATKPPTLLTDLPRYPWNYSTKYWQESRTTHKHKNRTAKRSDILGVEAIYSSDLEPTWRNIVRLDDLPWLRHHRIQSLNVFPMSAFLVMALEASAQHASKKSRTVESFELRDVTVVKPLIVPDEDIEMTITLRPHQESGSSLLPASASDANLCQFRISSFTQAKGWTEHCIGLVGTQTTEEKTNDVDSVRQASTAAAAVRYAKLLVGDKSGSAVNSAKMYDALDKLGVSFGPTFQGVENCRATDNAACADLAVIDVANEMPSHYLSSPSLHPSFLESLIELYWPILGAARTETGVRNVYLPASLERLTVSAKAIELSQKPGNLLSAYCTAEDIASAVEGEKSAKVTLYATADRESSESLITLENLVISPMIEGESGLDDGANNARELCYKLEWEPVLEAAEPVEGTEPAAPGFPDAKVVIIHEDNQSQQAIASGLSATLEKATGVTPETGSLETVDVAGKICVFLSELSRPVLSTLTESEFNALKSVLTTAEACLWVVRGAYSKSSNPDANMILGLSRVIRSESAMKFATLDLDATHVLPEDETAAAVARVFEAAFGATASNTSDLEFSEQNGRFFTPRIIDDADMNDYVHRQTHPDILEPTVFGANGRSLKMTLGRSGALDTVHFVEDLDLAQTDLAADEIEIEVKAIGVNARDLATATKGQVSSMGIEASGLVKAVGTAVTQFRAGDRVAALTPSHGSFATRTRTTATLAFKLPASVSFEAGAALPLAYVTAYHSLIELGRLAEGDSILIHAASGAVGQAAICLAQMIGADIYATVSSADKKALLAEEYNIPESNIFYSRDNSFGRAVRQATGGQGVDVVLNSLSSAQAVRESWATLNKFGRFVDIAPRDTSSSRRLLEMSSTDASNASYMSVDIFALASERPKTVRRLVADVSSLLKYGKIRPAAPITSFPISDVENALKAVHSSAATRTPGKMVVVPHAEDVVRATASLNQMSDLLKADGTYVLVGGTGGLGRSMARWMSKRGAKHIVLVSRTGSATGEVKTLIDDLASETGTNVYVHQCDVVNRKSVDNLLAVGLSGLPPVRGIVHGTMVLRDVLFEKMTFEDYDKVIEGKVRGGWNFHHALNETKAPLDFFVAISSAAGAVGNRGQGAYAAANCFLNALVQLRRAAGQPASSLDLTMVSDSGYLANNADKLAEVARNLGSDSICEAEVLALLGAAITGRLDETCEGHTITGMRITPSMQPFWTQDAKFKHLRIAMEEQAAAENAANGGAVAVSFNALLKAAQTLPEAEDAVAAGLVDKIAAVLMMEPEDMDVTRSLSHYPLDSLVAIEIRNFITREFEANMQVLELLSSGSIQTLSKAVCAKSKLVSFS

**Predicted cluster (antiSMASH prediction):**

| OphioH327gp6968 |
| --- |
| OphioH327gp6969 |
| OphioH327gp6970 |
| OphioH327gp6971 |
| OphioH327gp6972 |
| OphioH327gp6973 |
| OphioH327gp6974 |
| OphioH327gp6975 |
| OphioH327gp6976 |

>OpPKS8 (OphioH327gp7312)

MGSLVTAAPKVAQEPIAIVGSSCRFPGGATSPSKLWDLLKEPRDIVQEIPPTRFDTKAFYHPDSQHHGSANTKYAYLLQDDPRAFDRDFFSISPKEAEAMDPQQRCLLETVYEGVESAGYSIPKLRGSSTGVFVGAMSFDYQLVAMRGIDSLPQYHATGGSMAILANRISYFYDWKGASVTFDTACSSSLVALHQAVLALRSGEVDMAVTAGSNLILGPEPFIAYSNLNMLSPNGRSYMWDSSADGYTRGEGFASIILKTLSQALADGDQIEAIIRETGVNSDGRTPGITMPSSEAQTKLIRDTYAKCGLDITSPRDRPQYFEAHGTGTPAGDPIEAQAIHDAFFPGGLSQTHNKLIVGSIKTVLGHTEGTAGLAGVLKASLAVQHGQIPGNLHFSELNAKIRPFYDNLQVPTALTAWPALPAGQPRRVSVNSFGFGGTNAHAIIESWDRSTRDIVVASTPKSREAGLFVLSANSAQALAAKAETLVTYLLQKPDTDLGRLSRTLFQKTEFLFRAAFSATSVTQLIEKLEAAADGLPKTSRTATIPECLPLRILGVFTGQGAQWATMGSDLYRQSAVFRTTLDRLQHSLDTLPEADRPDWTLINELSAPKETSRVGTAVISQPLCTALQVALVDTLRAVGVEFAAVVGHSSGEIAAAYAAGYLDDRDAIRVAYYRGLFSHLAQGPDGKRGKMMAVGQSLEQAYGFCSQFGNAIKVAANNSPTSCTLAGDAGAIDKAKEMLDETNTFARVLAVDTAYHSHHMLLCAAPYLEALSKCGIQPLKGGNKCTWYSSVWGANGYSRSLNDDAGLKLLAGQYWVDNLTNTVQFSQALSRAVTDEPWIMDVALEVGPHPALKGPSSEAIKTVTGIAVPYSGVLKRGEGAFEAFANALGLLWTSFPSSRPLITFDKLHRALSQVSPKQYPSILKDLPSYPWDHDSLIWRESRASRSFRTQSRPRHELLGFPVTVGEQSRREVHWRQIFKLNELPWVRGHSIQGEVLFPATGYLTMAYEAAVQLVEDQQMLQLVELHDIEMVRAMSLQEDSPGLEIIFTVRVTSQSDSLITAEVACYSGDVSLAKLDGPLSGLSSHFTGGVRLTLGKPTKDALPTRAEPLLPMNTLDTDGFYSYLSSVGYNYSDPFRASTVDRHLNQAVVTVPIAPQPTSIRPSLHPTVLDTAIQGLLAGFSYPEDGRLKAVYLPTGIDCVRINMEPDAPGVLKADSFLTSADAKSLSGDVDLFNPDNSTVIAQMRGVHWTSLNQEGSRWLYAGETWLRDAAYGIEPGLKTTLSAEDEELRTLLIRTAFFYLRRLRDQIKPSELLIMGKHRRHMMKWIRDHLFPQIEAGKHPDVQKDWVNDTLEDVQKWSARYLDAGNNDMQLLHAVGSKLPAIARGTLPPLQVLLKDGMLDRLYVEGVGFSDGNMDLEVVAKQLAHQHPRMKVIEIGAGTGGTTRAVLSAMGTQYTSYMYTDISAGFFESAKTKFEQHVGQMTFKMLNIEKSPADQGFEEGSFDMLVASNCLHATRSLRDTLRHCRRLLRPGGQLLLLEITQDHLPIQLIMGTLPGWFLGAEEGRIWAPTISLAEWDAVLKETGFTGVNTSSTPSFCSVILSQATDETFQAIYEPLSVPAPSTALPRDEILVIGNSELASKSEAALAAAGATVIQSSLDSTKIPSGAVVLCLSELDRPVFDGMTQERFDAVQNMFRQASAILWVTSGAQSGKHPLANVTVGLGRTLLAERIDLRLQFLDVDSPESLEPSLLAKLLLQLTVAPPSELDDGILWTNEPHLSLRDGAMYIPRIKALDTVNYRSSARNKKVTQSTSLEIGDSAVEIAERNGSLEFVNVDIGGAREGELRAKVTASSVFTLLCDASPEHLWIGHDIKSGESIMGLSDANRSIVAASDDHVLHRWNAPNDSKQISDAAQLHGFMAHALAKHLLNNVVDSVWIHGAPNYLSQQIAWVAKDEGLVVFQTTSDTASAGSANQTSIHPYSSARDLQKLVPRGVKSLININPLRNKSLAALLRASVPRSTPVTELEDTAIRLTREELRKLAKKYLSGTITTSDQLEQILPVNDVSTETVKGLGPAAVIDWCASKEVTTVVRPIEYSSLFHADKTYLIFGMTGDVGISIAGWMAENGAGNIVLASRAPNVPAGVLEFMAQKGANVRIMAVDIANKQALVAACDDIASSLPPVAGVINGAMVLRDRLFVDMSWSDFEAVLAPKVTGTQNLDDLFGQDKNVDFFIVLSSATSMVGIMGQSAYSAANHFGASLVKQRRARGLAGSVVVIGFLTGLGYIFRRSDKAHLATIEKSLLPRLARQSETDLHEMLAEAIVCGQPGSGLPAELITGVRTAFQDTWHNDPRLSCYLVQENVQESTGQEEANGNVSVEVQLAASEDPKESLAILNKCFSQALGNMLQLDASKISVDEPAVSLGVDSLVSVRVREWFLKELGVEVPIMKIMSTNHSLSRLCEDALSGWRKLKGAPNAAAPAAPTTRDPEMDWAKELADVINGIPAIIPSGADLVKDAPRTTDRRVVVTGCTGFLGTHMLRSLVEDPSVAEVNCLCIRSRHVRVQSPKIHEYKGDLLKPLLGLSAADFVHLSQTADVIIHLGAEVNHLKSYGGMRTANVISTQILLAMATPRSVPVHFVSSSSVAMLQKDTSELPEIPPSQIKPPTDSESLMMNAIGYAASKWMGEMLLEHAAPPAVVHRFPNIMGPDAPAEIPLVALDRWCKKMRAVPALDPKQWVGQLDIIEVSDVVPEFIANAFAHDPSKSFAVINYCSENEYWLSDLAGMYEKTIGGPIEVVPTELWMKRATATGMPKTVVTTFTGHDEVFVSPVLRKGPRKA

**Predicted cluster (BGC boundaries were delimited by comparative genomic analysis):**

| OphioH327gp7305 |
| --- |
| OphioH327gp7306 |
| OphioH327gp7307 |
| OphioH327gp7308 |
| OphioH327gp7310 |
| OphioH327gp7311 |
| OphioH327gp7312 |

>OpPKS9 (OphioH327gp8463)

MRPEVEQELAHTLLVELLAYQFASPVRWIETQDVFLADKTAERIVEIGPADTLGVMAKRTIASKYEAYDAAKSVQRQILCYNKDAKEIYYDVDPVEDEPEPTPEASSSAAPAAAAAGAPAAAAPVAAPAPSAGPAAAVADAPVQALEIVRALIAQKLKKPLLEVPLSKAIKDLVGGKSTLQNEILGDLGKEFGSTPEKPEDTPLDELGAAMQATFDGNLGKQSQALIARLISSKMPGGFNITAARKYLESRWGLAQGRQDGVLLLAITMEPPARLGAEGDAKAFLDNVAQKYAVNAGISLTTASAAGADAGGGGGMMMDPAAIDALTKDQRALFKQQLELLARYLKMDLRAGDKAFQTSQESSKLLQSQLDLWSAEHGDFYAAGIEPAFSSLKARVYDSSWNWARQDALSMYYDIIFGRLQAVDREIVSQCIRIMNRSNPTLLEFMQYHIDNCPTERGETYKLAKELGAQLIENCRDVLNVAPVFKDVAVPTGPRTTVDARGNLNYEEVPRASCRKLEHYVQQMAAGGKITEYGTRAKVQTGLSRLYKLMKQQHKVSKTSQLEFKQLYGELLRSLEMNESQIISANGAAKKSLSSKPSGTPKAKVETIPFLHLRSQAMSGWDYNKKLTGLYLNCLADAAAAGVSFQGKYALMTGAGAGSIGADVLQGLISGGAHVIVTTSRYSREVTEYYQAMYARHGSRDSQLVVVPFNQGSKQDVEALVQYIYDPKNGLGWDLDFIVPFAAISENGRQIDGIDSKSELAHRIMLTNLIRLLGSVKTEKANRGFSTRPAQVILPLSPNHGTFGSDGLYSESKLGLETLFNRWHAEDWADYLSVCGAVIGWTRGTGLMSGNNIVAEGVEQYGVRTFSQQEMAFNLLGLMSPAVVDLCQNEPVMADLNGGLQFIPNLNEIMTKLRKDILQTSEIRKEVSKENSIENKIVNGEASEAFYKKKVIEPRANIKFEFPNLPDWKQEIEPLNAKLQGMVDLEKVVVVTGFAEVGPWGNSRTRWEMEAYGEFSLEGCVEMAWIMGLIKNHNGPIKGKPYSGWVDAKTGDPVDDKDIKNKYEKFILEHSGIRLIEPELFDGYDPEKKQLLHEVVIDEDLEPFEASKETAEEFKREHGEKVEIFAIPDSDQYTVRLKKGAGLWIPKALRFDRLVAGQVPTGWDAKRYGVPEDIISQVDPVALFVLVSVAEALLSCGITDPYEFYQYVHVSEVGNCIGSGMGGTAALRGMHRDRFLDKPLQNDILQESFINTMSAWVNMLLLSSSGPIKTPVGACATAVESVDIGYETITEGKARICIVGGFDDFGEEGSYEFANMKATSNAVDEFAHGRTPKEMSRPTTTTRNGFMESQGSGVQVIMTAQLALEMGVPIYGIVALTTTASDKIGRSVPAPGMGVLTTARENAGKFPSPLLDISYRRRQIEHRKKQIAQWKESELEFLADEIAAIKAQSDESFDEKAYTADRVNHVEKEAARQVKEVLRSLGNNFWKGDTSIAPLRGALATWGLTIDDLDVASFHGTSTKANDKNESSVICQQLSHLGRKKGNALLGIFQKYLTGHPKGAAGAWMMNGCLQVLNTGLVPGNRNADNVDEVMEKFDYIVYPSRTLQTDGVKAFSVTSFGFGQKGAQAIGIHPRYLYAALDEATFNAYRTKVEARQKKAYRFFHNGMINNSVFVAKTHAPYSDEQLPSVLLNPDARVNTDKKTAELTYPANFAQLANKAVPNAKSRTTLEKLQVLASEIETAHQNNKVGIDLEDISAINIENDTFLERNFTEAEIAYCNSTPNVQAAFAGRWSAKEAVFKSLGVASRGGGAPLKEIEVLRSETGAPTVHLHGEAASAAKAANLKEVSVSISHSDSQAVAIAVSTFA

**Predicted cluster (Backbone gene predicted by SMIPS/CASSIS was unable to predicted BGC boundaries)**

>OpPKS10 (OphioH327gp7900)

MTSSNSFGDLGLSIIGLGHHYPPHSLKPDALETLAKRFYPDSPAMKKVLAINRYTGIDQRASIGTPDHPLVNQENAPSIAELNAIFFEEGVPLAVRASELALAEAGLAGAGMSQITHIVSATCTNSANPGFDHYVAKGLGMTHQVEKVLLHGIGCSGGLAALRTAANLALGHAMRGKPARILVVTLEVSTTMVRSELDSIDESQETRIGVALFSDCGSAVVLSNGISLANKTEPAAEPIYELLGWDHRIIPDTEQDLGFDVDAVGWKVVLSPRVPTMAASQLQPAFADLMASTRKMLPPRGDYQKPADFDWAMHPGGATILSGAERVLGITPHHMRASYDTYMKHGNSSSATIFSVLDRLRDEDMDKLAVGGEGQPRKYVIGCAFGPGISVEACVLKRNLHRAPRASSASSASSASAVRGATGLVTPPETESEASRSEAGDEVAAVPTAAAPAAPPTTEQFISSAINDLDLD

**Predicted cluster:** Comparative genomic analysis suggests that this type III PKS may not grouped in a biosynthetic cluster.

>OpTERP1.1 (OphioH327gp7267)

MTRDALSIDIDRPYVIISEGVTTPTSPRSVGMPLLKQPEMLYLPNTLADWPWSRVLNPHYNEVGQQSREWLRSFNAFPTCAQNAFDACDFNLLASLAYPRHSKDHVRIGCDVMNLFFVIEEYTDDASTKDTARVVNMVMDALRHPHTPRPEDEKRTCLAGEVSRQFWENAVRTSSKSSQARFVAAFDAYTQGVIREAQDRNTHVGHIEHSIDSYLQLRRQTIGCWPSFALLGLNTEHLTDNVMNHPLVEALGHLATELILMGNDLLSYNRERKDGALHNLVTIVLSNPPSTHPYISTPQEALNWIFQYHDVLAKHFVLIYDAIKYMFEPGAPHHAQSVRLSAGSAVLPVDTTMTTTLTLVADSAESTQQASFSDDEKAALLDYADGLGNWAGDATTWRTNTLDDENDVAEILKEDELGPPTAVRRQGVSNLSMNGVGK

>OpTERP1.2 (OphioH327gp7266)

MLLSSTLGFWTGILMTILLLVVTYKLYLYPEFVSPLRNLPGPKDHHFALGQHLNQFRSGDPEEPYLSWMRKWPQAKMIRYYEFLHGDAILINNPSVYRSVMNDNAYAFVRSTPFRRLIGDIIGEGLVFAEGDAHRAQRRALGGLFTNTRIRDYVPDFNRKAMQLVIDVDNTARKYGKVDIKALFAEITLDVIGIFAFGIDLSQTKSRKDFVDCYTSMFDLTPGGMLLAAINLLVPIRWLPLKENAAFMNASVKLRQMMTQMVDTRIVEMGERNEKGKLYDSRGQLKSLDMLTHMIETRYQAAHDPWTKADLVEQALNFLATGHETTAGALTFAVHLLGQYPDVVKKIRAEAKNASITACDFEMSDEWGGGPTFNQIDSLVYLDHVLKECLRLLPPVAGIPRVATKDLIICGQLIPKGTTVMPVPAVIHHNPAIWGPDADEFKPERWEHAAGGAVEAEHFAWVGFGHGPRACIGRALAALNVKIVLLQIAVRFDFRPVNPGKVPVVNPSGQIRPLGEVFMYVRRAKNVFVFDQKQGTYHCLDKEGY

**Predicted cluster (antiSMASH prediction):**

| OphioH327gp7265 |
| --- |
| OphioH327gp7266 |
| OphioH327gp7267 |

>OpTERP2 (OphioH327gp1735)

MPSLKDVAYLLVHPNQLRSIIQWKLWHDPVHVRDPSKESATEKACFQYLDLTSRSFAAVIKELNPELLMPVCLFYLALRGLDTIEDDMTLSNERKIPLLRNFDSLMEQDGWTFTENGPNEKDRELLVNFDKVIVELKRMKPEYYTVVRDITAKMGNGMADYALNAEHNTNGVNSVADYELYCHYVAGLVGDGLTRLFVEAKLANEALLERPALTESMGQFLQKTNIIRDVHEDFLDKRRFWPKEIWSKHVDNWDDLFNPAPEFRSKALACSSEMVLNALKHADECLFYMAGIRDQSVFNFVAIPQSMAIATLELVFQNGAIFESHIKITKGDACQLMSESTQNLRVVCEVFRRYLKKIHKKNDPRDPNFTAISLQCGKIEQFIESIYPTQDIKEIKAKEKASADALPLADSLVLVGCVIGVLVVVGGIMIGAAWFLGEHMDTILDYAGIASPSLAAGAAAVHEEL

**Predicted cluster (antiSMASH prediction):**

| OphioH327gp1733 |
| --- |
| OphioH327gp1734 |
| OphioH327gp1735 |
| OphioH327gp1736 |
| OphioH327gp1737 |
| OphioH327gp1738 |

>OpTERP3 (OphioH327gp0834)

MSSSKQKEKEPAAMITSNSTPPFMHSPNAIPPRRSSTSQQHQYQHHQHQLSQSSTSPSSSTSTPAAPSTSLSSASPSASTSISTPSAPPRQHRHHHSSSSTSNSSKAVNMLKAVPESDWMNNPAATRRPRKPSVTMASLAAADKPDRGTTPIVDLALPLPPQLVLQQQQQQQQQQQLHQKQLYLGQFTATASPAASVLNNSLQPTPPPDPSRYATEDFNFSARRTWTEEKERIVCGPYEYLNSTRGKEFRTQLISAFNGWLEVPEDSLEVITRVVGMLHTASLLVDDVEDSSSLRRGLPVAHNIFGVPQTINSANYIYFMALQELQKLHNPKALVIFAEELCNLHRGQGMDLFWRDTLTCPTEDDYLEMVGHKTGGLFRLGIKLMQAESRSLVDCVPLVNLLGLLFQIQDDYRNLMASEYSQNKGMCEDLTEGKFSFPVIHSIRTSPSNLQLLNILKQKTSDEEVKRYAVAHMEHTGSFAYTREVLEILTSRARNLADALDEGKGRSRAIHAILDKLVIN

**Predicted cluster (antiSMASH prediction):**

| OphioH327gp0831 |
| --- |
| OphioH327gp0832 |
| OphioH327gp0833 |
| OphioH327gp0834 |
| OphioH327gp0835 |

>OpTERP4 (OphioH327gp5830)

MRLACLRGIRPLHPGCMTLRSPASASLRMGKALSSSLATSSSSRRNSFFPVKASTVRISGPPSLIRLHTTAAAATAVASAEEEVAQPPYTPPTTGVIAMLPKSWIPYAELTRMDKPTGSYYLFFPCLFSTLMAAPMVEPAMTSPVSVAGTTLLFLAGSLIMRGAGCTINDLWDRNLDPHVARTRLRPIARGAVTPFNALVFTGAQLFAGLGILLQFPTSCLYYGIPSLAFVATYPLFKRITYYPQAMLGLTFSWGAIMGFPALGIDLLSNSAALATSAFLYTSNVAWTVLYDMIYAHMDVKDDVKAGIKSIALKHGAQTKAVLTGLGAVQIGLLTAAGVASGAGPAFFIGSCGGAAVTLGVMIKRVKLESVKNCWWWFVNGCLLTGGVISAGMGVDYVIRYRQQQQAEKAKLAITA

**Predicted cluster (Backbone gene predicted by SMIPS/CASSIS was unable to predicted BGC boundaries)**

>OpTERP5 (OphioH327gp1753)

MPPPGHLLLLRSSVGGRLRAADALIECCRRIGSLQTSQLRQLRYFSTPITSPTPTTLRKSAPHQHPAISPCRPFSSTSLRPSSAAAGSPGPRSSSSKSSSAPSSSAPPTNTSTLEERLKPSYFLSNTILDRIAGLSPGSALPSAAEALPHRRRKARRQAQLGASTASTPSSSSSPTQSVASAAELPANASTILTDMAASQTSVRRLASSLLSLAKPRLTVLVVLTAMAPYALYPVPSFLSPELIDAPSLSPLTLLFLTTGTALCSASANALNMLYEPDTDSRMSRTRNRPLVRSLVSRRAALLFAIGCGVVGVAALEWGVNPTTAFLGAANIALYAGVYTPMKRLSVLNTWVGALVGGIPPLMGWTAAAGEADNPREFRELLFAPDGSSAGGWLLAGLLFAWQFPHFMALSWAVRHEYQRAGLRMLAWVNPARNGRVALRYSIAFVPICVGLCAAGVTSWSFAATSLPVNLWLVREAVKFWRFEGHQGSARALFWASVWHLPALMVLALLQKKGMWTRVYRSVMGEPAYDDDDDDEFYDDDEEVDEEPLVGTPAQSKPVPSRASR

**Predicted cluster (Backbone gene predicted by SMIPS/CASSIS was unable to predicted BGC boundaries)**

>OpOTHER1 (OphioH327gp5165)

MSDITPDVVADRIARFGKCYTIDDVVRLRAKDPEQTPIIGVPRKMDDPGDYEYFTAADLDRMIDESCRILVKKGLAVAANSDIIIYGSSARIASTLAEVAKERPSVLMLPILTREEYDKPDAPAQPPFIREISDRDAEHAELAMMAHSSGSTGLPKPLLVSHRSLMNSLVLGTGLKAFNTLPWYHIHGLITSLQAMWMRRPTHLFNPYLPLTAANLIAALREIQPEICHGVPYALSLLAEDAAGVEVLKKCRFVTSGGAKTPDELGNRLVKAGVNLGVIYGLTEVGHVGDSIGREPGQSESWAYMRPCANIREHITFKHVEGDKYEAVYLESHPALLMSNSNNPPGSYYSNDLFTPHPTIPDAWKYIARLDDRLTLITGEKILPLAMEDAVRDHPLVKDALMFGNDRALPGMFVFRAKDAAAMTENEFLNAIWPSVERANAIADEFARITRETVASIAFGVEIPVTDKSNIIRAAAYRMFAKQIDAVYARLDGTSINGNSASIPKPDGPLDEAKLTSFVIDVFKTQTGIELPSAKSDFFASGVDSLRAIQARRIFQETLGFDGFKLPTNVVYDSRNAANLAHYFHGLLQGNGISANGNGNHTSGINSTNGINGVSVDNDDLAIMDSLVSKYSKFDTEIPAEHVGGESVLLTGATGALGAHVLHQLLHNRNVDRVTCLVRGPHGLKRVHESLAARDLSIRSNRKLARTLAVLETSNLGDEHLGLSSFDYNGLVADTTIIIHAAWPVHFGLSASSFEPHIAGLYNLLQLSLHVPSRHPARVIFASSISAAFNVPRPADGSPAPIPEGPLESYSDSSPMGYAQSKLIGERVCEAAAHQGANVAVLRIGQITGDTEHGIWNDREAIPLLVNSALELKALPLLGQETGRCEWTPVDVVAASCIEIAGSMKVGNALNRINGVNGHHKTNGVISGDSPFKARYYNINTPHVLSWNDDVLPEFKKAGLEFDTVSLGEWVDKLRARGEILGAEAAKRLPALKLADYYAKNFTDSEVAGIRFEIGKACSDSPTLGSCPNIAETQLVGKYVQSWLKAWTKK

**Predicted cluster (BGC boundaries were delimited by CASSIS):**

| OphioH327gp5163 |
| --- |
| OphioH327gp5164 |
| OphioH327gp5165 |
| OphioH327gp5166 |
| OphioH327gp5167 |
| OphioH327gp5168 |
| OphioH327gp5169 |
| OphioH327gp5170 |
| OphioH327gp5171 |

>OpOTHER2 (OphioH327gp5448)

MDTTHDLVELFRRQVLATPNAVAVVDDTSSWTYAELDKATDHVADRLRSVYGVGRDALVGVLMGHNIQYIVACMAALKAGGAFLVLEVAYPPGLLADVIEDALPPVILTSSEYADRVPVHTPRILLERGMTVADLAALPTSDPLITHSEPASPLPAATDLDRLAFVSYSSGTTGRPKGICNPHSAPVQSYALRFKISDLGPGDRVACNVFFVWEILRPLLKGGAVVTVPDDSSYDPVGLVKFLSQHEITETLMTPTLLATVLARYPDLATRLPHLRTLWLNGEVVSVDLARRATAALSPPGASPVRLLNCYSVSETHEIACGDIRVALEHLPSDATVCPVGPPMDPEHTYVLDEEGNLLPPGAGGELYVGGPLLARGYLNRPETTAEVFLPNRFAGGVGRMYRTGDKARILPSTGELEISGRVGGMIKVRGYTVHPAAVESAIVKHLAVRSCVVLADKEGLERQLVAYVVRDVEGGAASGPDLRSPLQINGDGHSPLARRLLADHLAHYMIPVFWVELDMLPTHKVSGKVDVKGLPSLRAAPSGANPGPSPPETPLADTLARTLWGNGSATVAPGGVQNDPATQIDENDVRRHWAACLNLPETSIDPQQFDFFDLGGHSLALAELSSRLSAAYGVNVPLLALASKPNMAGHIAAIAEAARAASTATVKSVDNSLLKAIIESDSVLPNELKATEKAKPARISEATDIFLTGATGFLGAFLLAELVATTKATIHCLVRGSSAYGHTKSGSGMARLRKHLLGLGLWSDTIVDRIEIVAGDLALPGLGLGSTQFDDLAKKVQAVVHAGAHVNLVYPYDALRDANVDGTKEVLRLAAQGKATIHYISTNGVLPPAKHAWNEDDTPISTPGDVVDKIPDGYGQTKWAAEQLVIAAAQQGIPARIYRPGTLGGHSNTGATNPRDVVTALIVESLRIGYSPNVDGWRIEMTPVDAAASKILSLADADADKTTDNSVLHIGDSDAILAKDLFALLTLLGYETQPVDWDEWVARWNEDQSDVPPASPASRDDGDLSPMDILRAGMPSVDFLKLVILLDDTKTNSLLEETEKHPSLDLELLTTYTRHFYSRGWLHKGPKLSVVPKVAESVVPVPKGPLAGRVAVVVGASSGIGAAIATALANDGAHIALGSRRLGELKTLQATLEAQFPGTKILPQATDVTDAAQVKALVEAAKTQLGPIDILIVCAGVMYFTMMSSVRSEEWDRTVDVNCKGLLHCLSATLPGMLERAKPINGASDGSGHSPHIVAISSDAGRKVFPGLGVYSGSKFFVEAVLQALRVETAGTGLRVTSVQPGNTATELLGLSTDPEAMEKYGAPTGAQVLSPADVASAVVYALRQPAHVSVNEVMIEPRDEPI

**Predicted cluster (antiSMASH prediction):**

| OphioH327gp5442 |
| --- |
| OphioH327gp5443 |
| OphioH327gp5444 |
| OphioH327gp5445 |
| OphioH327gp5446 |
| OphioH327gp5448 |
| OphioH327gp5449 |
| OphioH327gp5450 |
| OphioH327gp5451 |
| OphioH327gp5453 |
| OphioH327gp5454 |

>OpOTHER3 (OphioH327gp1586)

MASIEASSVLPDPTADLHWNEYRGAIHEIFNKNAHTHPERPCVVETKGPRTPERIFSYQQINESSNQLAHFFLAHGCERGDVVMIYAHRGVDLVVAYMGALKAGATVSVLDPQYPADRQKVLLEVASPRFLVQIQRATEEAGPMAQTVADFVAANLQIKAEVPALELDKDGKLFGGKLPGADVDCLAAQESLRPSFPDVQIGPDSTPTLSFTSGSEGKPKGVRGRHFSLTHYFPWMAQRFGLSENDRFTMLSGIAHDPIQRDIFTPLFLGAQIIVPPRDSIAHELLAEWMHVNKVTVTHLTPAMGQILVGGASTQFPSLHHAFFVGDLLTKKDSRRMQDLAPNAFIVNMYGTTETQRAVSYFEIASRNTDAAALDSLPDIIPVGQGMVNVQLLVVDREDRNRICDVGEQGELFLRAGGMAEGYLGDDEVSNKLNTEKFLTNWFTKPETWTVPYEKLAAEKPEPWMPYYKGPRDRIYRTGDLGRFRPDGTVECTGRVDNQVKIRGFRIELGEIDTHLSRHPYVRENVTLVRRNQDEEPTLVSYIVPESKRWLQNLADGGVSVEDQLLSVSNTPDESMSSMLKRFKLLSEDCKTFLKTKVPYYAVPTMFIPLARMPLNPNGKIDKPALPFPSDADLATLNRRASRASSALASMTETQKRLAALWAQVIPHRSARMFVPESNFFEEGGHSILAQRMMFLVRKEWKDIDVPMSAIFQSQTLGGFAAEIDRAQDPTGLRLDFGTGSAVGDDDAYAADARDAAAHMPEKIPSVETLGDGPTTAFLTGATGFLGIYVLRALLAQNAGNKVIALVRAKDTAAGLARVESIAKAYGLWDDSWAGRVEVVLGDISQPKLALDAVDWDRVASAARIVVHNGAMVNWMLPYSSMRAANVGSTLECIKLCATGAAKRLVFVSSTSTLDTDHYVQLSQQSNGVGILESDDLEGSRKGLGTGYGQTKWASEYLVREAGKRGLVGTIVRPGYITGNIESGISITDDFLIRLWKGCLQVGARPDITNTVNQVPVTHVSRIVASSAFNPPTVHPLSVVHITSHPRLTMNEWLGALEVYGYSVPQMPYRDWCSALRAYVADETQEEHALLPLFHFVVGNLPGDSIAPEMDDKNASAVLTATFGADAVPTSFPDAGVSTEAAGYKGPAARPLPAVSLSAEQLAQLANIGTRTTAKS

**Predicted cluster (antiSMASH prediction):**

| OphioH327gp1581 |
| --- |
| OphioH327gp1582 |
| OphioH327gp1583 |
| OphioH327gp1584 |
| OphioH327gp1585 |
| OphioH327gp1586 |
| OphioH327gp1587 |
| OphioH327gp1588 |
| OphioH327gp1590 |
| OphioH327gp1591 |
