# Supplementary material for: Genome-Wide Analysis of Secondary Metabolite Gene Clusters in Ophiostoma ulmi and Ophiostoma novo-ulmi Reveals a Fujikurin-Like Gene Cluster with a Putative Role in Infection
Source: Front Microbiol. 2017 Jun 13;8:1063. doi: 10.3389/fmicb.2017.01063 (PMC5468452; doi:10.3389/fmicb.2017.01063)
Supplement: Supplementary file 7 [file Image_1.PDF]

Supplementary Figure 1

Genome-wide analysis of secondary metabolite gene clusters in *Ophiostoma ulmi* and *Ophiostoma novo-ulmi* reveals a fujikurin-like gene cluster with a putative role in infection

Nicolau Sbaraini [1, 2], Fábio Carrer Andreis [1, 2], Claudia Elizabeth Thompson [1, 2, 3], Rafael Lucas Muniz Guedes [1, 3], Ângela Junges [2], Thais Campos [2], Charley Christian Staats[1, 2], Marilene Henning Vainstein [1, 2], Ana Tereza Ribeiro de Vasconcelos [1, 3], Augusto Schrank [1, 2,*].

*** Correspondence:**
Augusto Schrank
aschrank@cbiot.ufrgs.br

Phylogenetic trees constructed with putative orthologs of OpPKS8 and all characterized PKS from MIBiG.

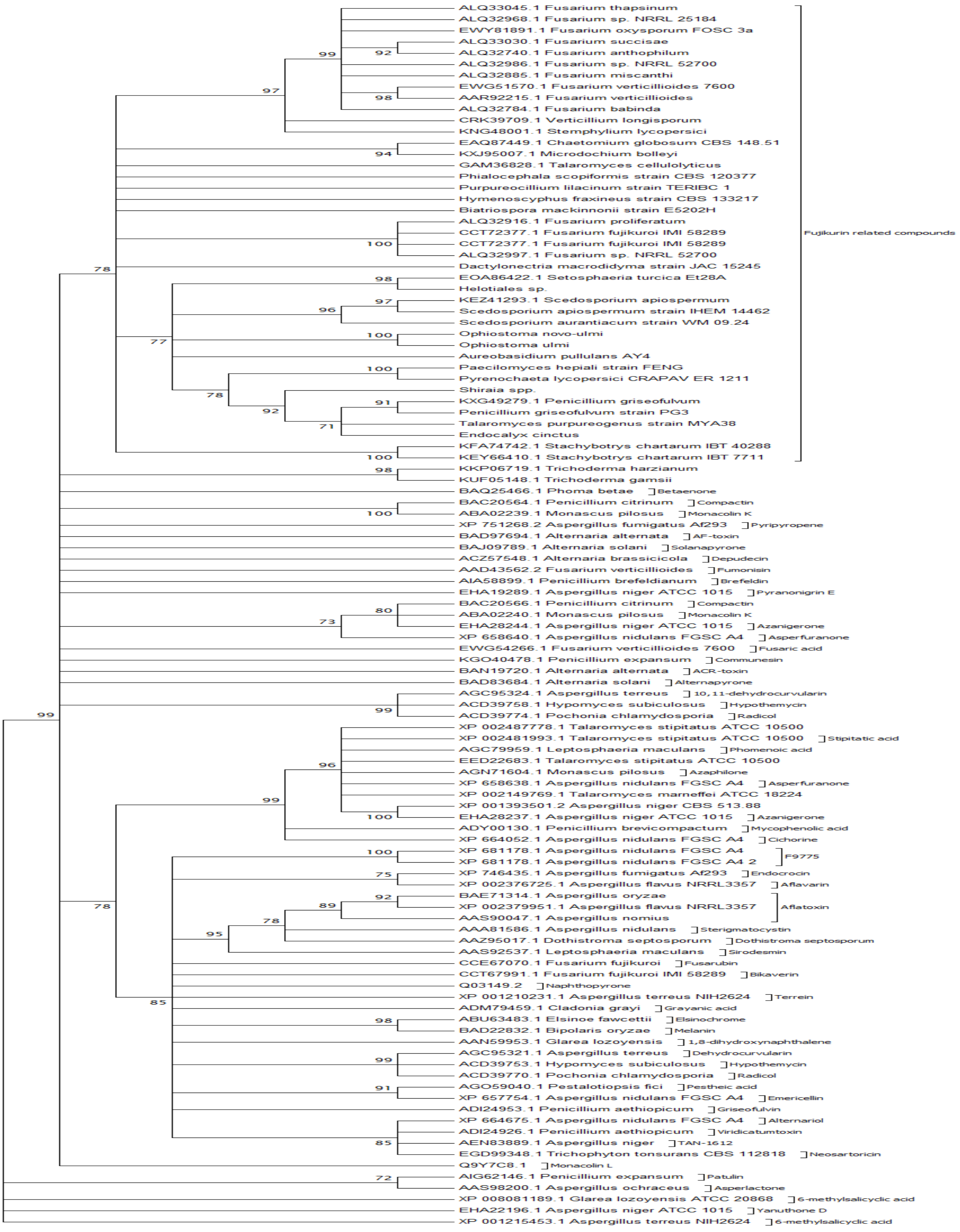

ALQ33045.1 Fusarium thapsinum
ALQ32968.1 Fusarium sp. NRRL 25184
EWY81891.1 Fusarium oxysporum FOSC 3a
ALQ33030.1 Fusarium succisae
ALQ32740.1 Fusarium anthophilum
ALQ32986.1 Fusarium sp. NRRL 52700
ALQ32885.1 Fusarium miscanthi
EWG51570.1 Fusarium verticillioides 7600
AAR92215.1 Fusarium verticillioides
ALQ32784.1 Fusarium babinda
CRK39709.1 Verticillium longisporum
KNG48001.1 Stemphylium lycopersici
EAQ87449.1 Chaetomium globosum CBS 148.51
KXJ95007.1 Microdochium bolleyi
GAM36828.1 Talaromyces cellulolyticus
Phialocephala scopiformis strain CBS 120377
Purpureocillium lilacinum strain TERIBC 1
Hymenoscyphus fraxineus strain CBS 133217
Biatriospora mackinnonii strain E5202H
ALQ32916.1 Fusarium proliferatum
CCT72377.1 Fusarium fujikuroi IMI 58289
CCT72377.1 Fusarium fujikuroi IMI 58289
ALQ32997.1 Fusarium sp. NRRL 52700
Dactylonectria macrodidyma strain JAC 15245
EOA86422.1 Setosphaeria turcica Et28A
Helotiales sp.
KEZ41293.1 Scedosporium apiospermum
Scedosporium apiospermum strain IHEM 14462
Scedosporium aurantiacum strain WM 09.24
Ophiostoma novo-ulmi
Ophiostoma ulmi
Aureobasidium pullulans AY4
Paecilomyces hepiali strain FENG
Pyrenochaeta lycopersici CRAPAV ER 1211
Shiraia spp.
KXG49279.1 Penicillium griseofulvum
Penicillium griseofulvum strain PG3
Talaromyces purpureogenus strain MYA38
Endocalyx cinctus
KFA74742.1 Stachybotrys chartarum IBT 40288
KEY66410.1 Stachybotrys chartarum IBT 7711
Fujikurin related compounds
KKP06719.1 Trichoderma harzianum
KUF05148.1 Trichoderma gamsii
BAQ25466.1 Phoma betae Betaenone
BAC20564.1 Penicillium citrinum Compactin
ABA02239.1 Monascus pilosus Monacolin K
XP 751268.2 Aspergillus fumigatus Af293 Pyripyropene
BAD97694.1 Alternaria alternata AF-toxin
BAJ09789.1 Alternaria solani Solanapyrone
ACZ57548.1 Alternaria brassicicola Depudecin
AAD43562.2 Fusarium verticillioides Fumonisin
AIA58899.1 Penicillium brefeldianum Brefeldin
EHA19289.1 Aspergillus niger ATCC 1015 Pyranonigrin E
BAC20566.1 Penicillium citrinum Compactin
ABA02240.1 Monascus pilosus Monacolin K
EHA28244.1 Aspergillus niger ATCC 1015 Azanigerone
XP 658640.1 Aspergillus nidulans FGSC A4 Asperfuranone
EWG54266.1 Fusarium verticillioides 7600 Fusaric acid
KGO40478.1 Penicillium expansum Communesin
BAN19720.1 Alternaria alternata ACR-toxin
BAD83684.1 Alternaria solani Alternapyrone
AGC95324.1 Aspergillus terreus 10,11-dehydrocurvularin
ACD39758.1 Hypomyces subiculosus Hypothemycin
ACD39774.1 Pochonia chlamydosporia Radicol
XP 002487778.1 Talaromyces stipitatus ATCC 10500
XP 002481993.1 Talaromyces stipitatus ATCC 10500 Stipitatic acid
AGC79959.1 Leptosphaeria maculans Phomenoic acid
EED22683.1 Talaromyces stipitatus ATCC 10500
AGN71604.1 Monascus pilosus Azaphilone
XP 658638.1 Aspergillus nidulans FGSC A4 Asperfuranone
XP 002149769.1 Talaromyces marneffei ATCC 18224
XP 001393501.2 Aspergillus niger CBS 513.88
EHA28237.1 Aspergillus niger ATCC 1015 Azanigerone
ADY00130.1 Penicillium brevicompactum Mycophenolic acid
XP 664052.1 Aspergillus nidulans FGSC A4 Cichorine
XP 681178.1 Aspergillus nidulans FGSC A4
XP 681178.1 Aspergillus nidulans FGSC A4 2
F9775
XP 746435.1 Aspergillus fumigatus Af293 Endocrocin
XP 002376725.1 Aspergillus flavus NRRL3357 Aflavarin
BAE71314.1 Aspergillus oryzae
XP 002379951.1 Aspergillus flavus NRRL3357
AAS90047.1 Aspergillus nomius
Aflatoxin
AAA81586.1 Aspergillus nidulans Sterigmatocystin
AAZ95017.1 Dothistroma septosporum Dothistroma septosporum
AAS92537.1 Leptosphaeria maculans Sirodesmin
CCE67070.1 Fusarium fujikuroi Fusarubin
CCT67991.1 Fusarium fujikuroi IMI 58289 Bikaverin
Q03149.2 Naphthopyrone
XP 001210231.1 Aspergillus terreus NIH2624 Terrein
ADM79459.1 Cladonia grayi Grayanic acid
ABU63483.1 Elsinoe fawcettii Elsinochrome
BAD22832.1 Bipolaris oryzae Melanin
AAN59953.1 Glarea lozoyensis 1,8-dihydroxynaphthalene
AGC95321.1 Aspergillus terreus Dehydrocurvularin
ACD39753.1 Hypomyces subiculosus Hypothemycin
ACD39770.1 Pochonia chlamydosporia Radicol
AGO59040.1 Pestalotiopsis fici Pestheic acid
XP 657754.1 Aspergillus nidulans FGSC A4 Emericellin
ADI24953.1 Penicillium aethiopicum Griseofulvin
XP 664675.1 Aspergillus nidulans FGSC A4 Alternariol
ADI24926.1 Penicillium aethiopicum Viridicatumtoxin
AEN83889.1 Aspergillus niger TAN-1612
EGD99348.1 Trichophyton tonsurans CBS 112818 Neosartoricin
Q9Y7C8.1 Monacolin L
AIG62146.1 Penicillium expansum Patulin
AAS98200.1 Aspergillus ochraceus Asperlactone
XP 008081189.1 Glarea lozoyensis ATCC 20868 6-methylsalicyclic acid
EHA22196.1 Aspergillus niger ATCC 1015 Yanuthone D
XP 001215453.1 Aspergillus terreus NIH2624 6-methylsalicyclic acid

Figure 1: Maximum Parsimony analysis of taxa. The evolutionary history was inferred using the Maximum Parsimony method. The most parsimonious tree with length = 2842 is shown. The consistency index is (0.352277), the retention index is (0.657330), and the composite index is 0.232910 (0.231562) for all sites and parsimony-informative sites (in parentheses). The percentage of replicate trees in which the associated taxa clustered together in the bootstrap test (1000 replicates) are shown next to the branches. The MP tree was obtained using the Subtree-Pruning-Regrafting (SPR) algorithm with search level 1 in which the initial trees were obtained by the random addition of sequences (10 replicates). The analysis involved 109 amino acid sequences. All positions containing gaps and missing data were eliminated. There were a total of 131 positions in the final dataset. Evolutionary analyses were conducted in MEGA6.

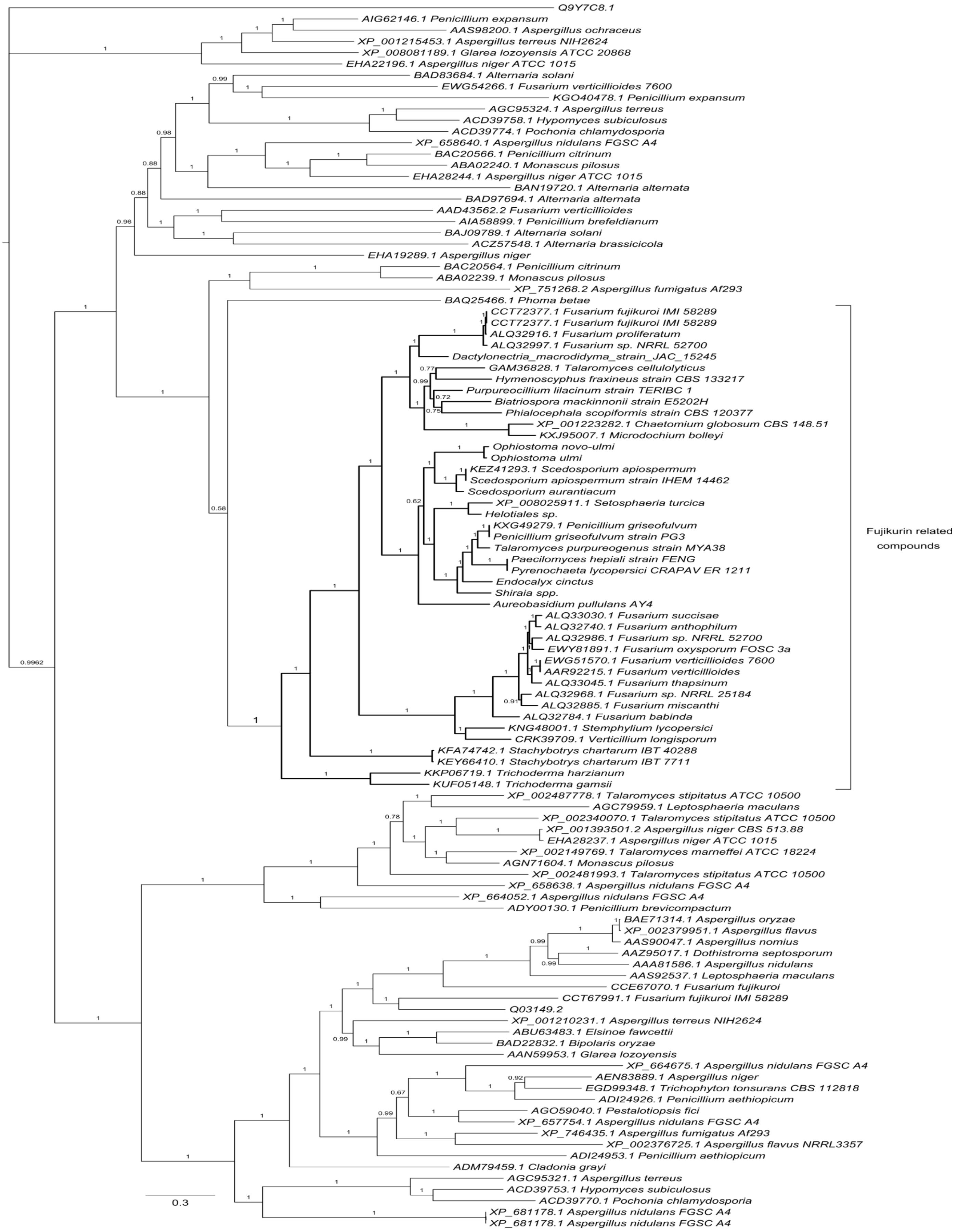
Q9Y7C8.1
AIG62146.1 Penicillium expansum
AAS98200.1 Aspergillus ochraceus
XP_001215453.1 Aspergillus terreus NIH2624
XP_008081189.1 Glarea lozoyensis ATCC 20868
EHA22196.1 Aspergillus niger ATCC 1015
BAD83684.1 Alternaria solani
EWG54266.1 Fusarium verticillioides 7600
KGO40478.1 Penicillium expansum
AGC95324.1 Aspergillus terreus
ACD39758.1 Hypomyces subiculosus
ACD39774.1 Pochonia chlamydosporia
XP_658640.1 Aspergillus nidulans FGSC A4
BAC20566.1 Penicillium citrinum
ABA02240.1 Monascus pilosus
EHA28244.1 Aspergillus niger ATCC 1015
BAN19720.1 Alternaria alternata
BAD97694.1 Alternaria alternata
AAD43562.2 Fusarium verticillioides
AIA58899.1 Penicillium brefeldianum
BAJ09789.1 Alternaria solani
ACZ57548.1 Alternaria brassicicola
EHA19289.1 Aspergillus niger
BAC20564.1 Penicillium citrinum
ABA02239.1 Monascus pilosus
XP_751268.2 Aspergillus fumigatus Af293
BAQ25466.1 Phoma betae
CCT72377.1 Fusarium fujikuroi IMI 58289
CCT72377.1 Fusarium fujikuroi IMI 58289
ALQ32916.1 Fusarium proliferatum
ALQ32997.1 Fusarium sp. NRRL 52700
Dactylonectria_macrodidyma_strain_JAC_15245
GAM36828.1 Talaromyces cellulolyticus
Hymenoscyphus fraxineus strain CBS 133217
Purpureocillium lilacinum strain TERIBC 1
Biatriospora mackinnonii strain E5202H
Phialocephala scopiformis strain CBS 120377
XP_001223282.1 Chaetomium globosum CBS 148.51
KXJ95007.1 Microdochium bolleyi
Ophiostoma novo-ulmi
Ophiostoma ulmi
KEZ41293.1 Scedosporium apiospermum
Scedosporium apiospermum strain IHEM 14462
Scedosporium aurantiacum
XP_008025911.1 Setosphaeria turcica
Helotiales sp.
KXG49279.1 Penicillium griseofulvum
Penicillium griseofulvum strain PG3
Talaromyces purpureogenus strain MYA38
Paecilomyces hepiali strain FENG
Pyrenochaeta lycopersici CRAPAV ER 1211
Endocalyx cinctus
Shiraia spp.
Aureobasidium pullulans AY4
ALQ33030.1 Fusarium succisae
ALQ32740.1 Fusarium anthophilum
ALQ32986.1 Fusarium sp. NRRL 52700
EWY81891.1 Fusarium oxysporum FOSC 3a
EWG51570.1 Fusarium verticillioides 7600
AAR92215.1 Fusarium verticillioides
ALQ33045.1 Fusarium thapsinum
ALQ32968.1 Fusarium sp. NRRL 25184
ALQ32885.1 Fusarium miscanthi
ALQ32784.1 Fusarium babinda
KNG48001.1 Stemphylium lycopersici
CRK39709.1 Verticillium longisporum
KFA74742.1 Stachybotrys chartarum IBT 40288
KEY66410.1 Stachybotrys chartarum IBT 7711
KKP06719.1 Trichoderma harzianum
KUF05148.1 Trichoderma gamsii
Fujikurin related compounds
XP_002487778.1 Talaromyces stipitatus ATCC 10500
AGC79959.1 Leptosphaeria maculans
XP_002340070.1 Talaromyces stipitatus ATCC 10500
XP_001393501.2 Aspergillus niger CBS 513.88
EHA28237.1 Aspergillus niger ATCC 1015
XP_002149769.1 Talaromyces marneffei ATCC 18224
AGN71604.1 Monascus pilosus
XP_002481993.1 Talaromyces stipitatus ATCC 10500
XP_658638.1 Aspergillus nidulans FGSC A4
XP_664052.1 Aspergillus nidulans FGSC A4
ADY00130.1 Penicillium brevicompactum
BAE71314.1 Aspergillus oryzae
XP_002379951.1 Aspergillus flavus
AAS90047.1 Aspergillus nomius
AAZ95017.1 Dothistroma septosporum
AAA81586.1 Aspergillus nidulans
AAS92537.1 Leptosphaeria maculans
CCE67070.1 Fusarium fujikuroi
CCT67991.1 Fusarium fujikuroi IMI 58289
Q03149.2
XP_001210231.1 Aspergillus terreus NIH2624
ABU63483.1 Elsinoe fawcettii
BAD22832.1 Bipolaris oryzae
AAN59953.1 Glarea lozoyensis
XP_664675.1 Aspergillus nidulans FGSC A4
AEN83889.1 Aspergillus niger
EGD99348.1 Trichophyton tonsurans CBS 112818
ADI24926.1 Penicillium aethiopicum
AGO59040.1 Pestalotiopsis fici
XP_657754.1 Aspergillus nidulans FGSC A4
XP_746435.1 Aspergillus fumigatus Af293
XP_002376725.1 Aspergillus flavus NRRL3357
ADI24953.1 Penicillium aethiopicum
ADM79459.1 Cladonia grayi
AGC95321.1 Aspergillus terreus
ACD39753.1 Hypomyces subiculosus
ACD39770.1 Pochonia chlamydosporia
XP_681178.1 Aspergillus nidulans FGSC A4
XP_681178.1 Aspergillus nidulans FGSC A4
0.3

Figure 2: Bayesian analysis of taxa. Branches support values (Bayesian posterior probability) are associated with nodes. The Bayesian inference ran for 528,000 generations.
