# Supplementary material for: Genome-Wide Analysis of Secondary Metabolite Gene Clusters in Ophiostoma ulmi and Ophiostoma novo-ulmi Reveals a Fujikurin-Like Gene Cluster with a Putative Role in Infection
Source: Front Microbiol. 2017 Jun 13;8:1063. doi: 10.3389/fmicb.2017.01063 (PMC5468452; doi:10.3389/fmicb.2017.01063)
Supplement: Supplementary file 8 [file Image_2.PDF]

Supplementary Figure 2

Genome-wide analysis of secondary metabolite gene clusters in *Ophiostoma ulmi* and *Ophiostoma novo-ulmi* reveals a fujikurin-like gene cluster with a putative role in infection

Nicolau Sbaraini [1, 2], Fábio Carrer Andreis [1, 2], Claudia Elizabeth Thompson [1, 2, 3], Rafael Lucas Muniz Guedes [1, 3], Ângela Junges [2], Thais Campos [2], Charley Christian Staats[1, 2], Marilene Henning Vainstein [1, 2], Ana Tereza Ribeiro de Vasconcelos [1, 3], Augusto Schrank [1, 2,*].

*** Correspondence:**
Augusto Schrank
aschrank@cbiot.ufrgs.br

Logical diagram depicting the step-by-step process and connections between methodologies described in the Material and Methods.

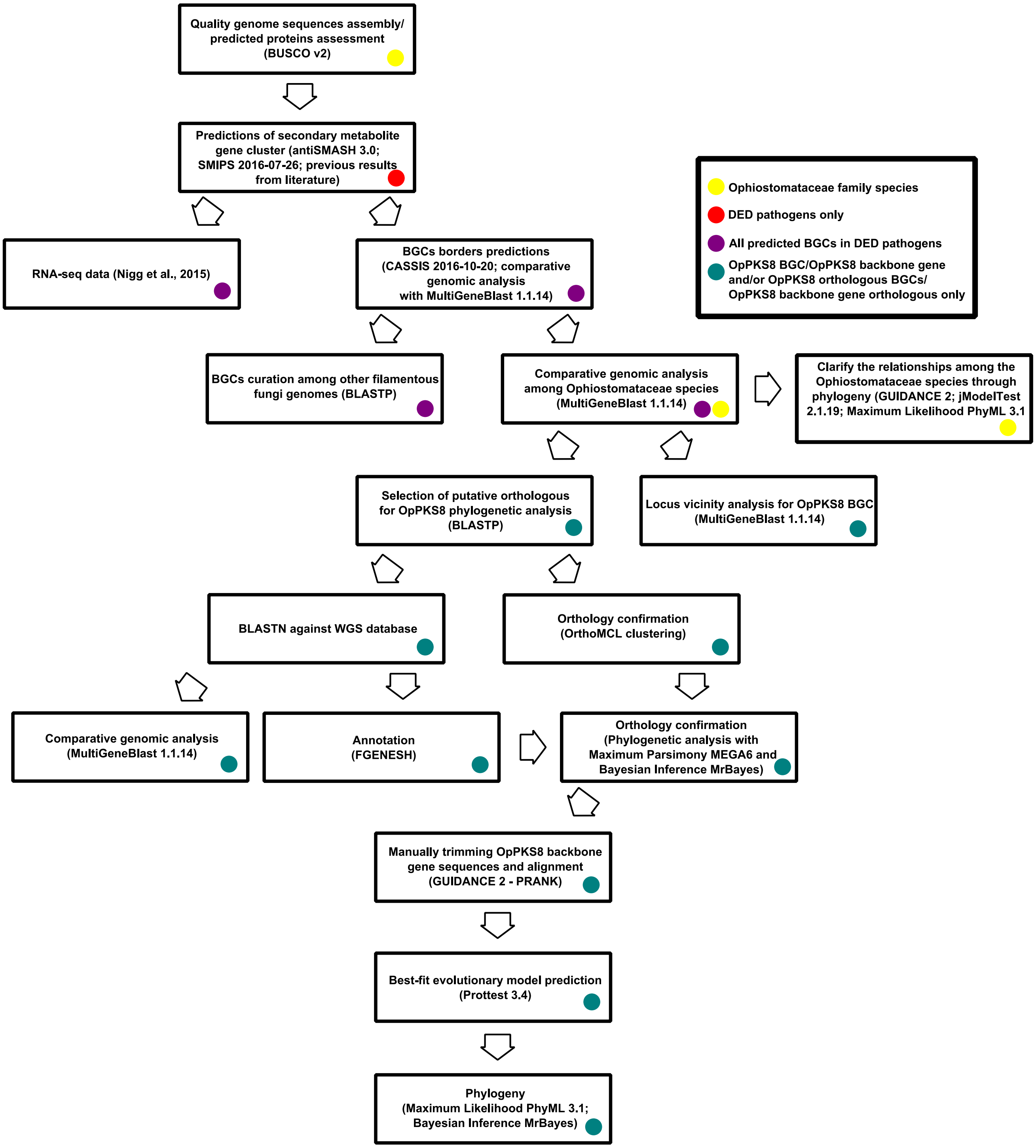

Figure 1: Logical diagram describing the step-by-step process and connections between methodologies described in Material and Methods. Colored dots represent the species/sequences/BGCs used in each step.
