# Supplementary material for: Genome-Wide Analysis of Secondary Metabolite Gene Clusters in Ophiostoma ulmi and Ophiostoma novo-ulmi Reveals a Fujikurin-Like Gene Cluster with a Putative Role in Infection
Source: Front Microbiol. 2017 Jun 13;8:1063. doi: 10.3389/fmicb.2017.01063 (PMC5468452; doi:10.3389/fmicb.2017.01063)
Supplement: Supplementary file 9 [file Image_3.PDF]

Supplementary Figure 3

Genome-wide analysis of secondary metabolite gene clusters in *Ophiostoma ulmi* and *Ophiostoma novo-ulmi* reveals a fujikurin-like gene cluster with a putative role in infection

Nicolau Sbaraini [1, 2], Fábio Carrer Andreis [1, 2], Claudia Elizabeth Thompson [1, 2, 3], Rafael Lucas Muniz Guedes [1, 3], Ângela Junges [2], Thais Campos [2], Charley Christian Staats[1, 2], Marilene Henning Vainstein [1, 2], Ana Tereza Ribeiro de Vasconcelos [1, 3], Augusto Schrank [1, 2,*].

*** Correspondence:**
Augusto Schrank
aschrank@cbiot.ufrgs.br

The species tree based on the sequences of the 18S ribosomal RNA gene (partial), internal transcribed spacer 1, 5.8S ribosomal RNA gene, internal transcribed spacer 2, and 28S ribosomal RNA gene (partial).

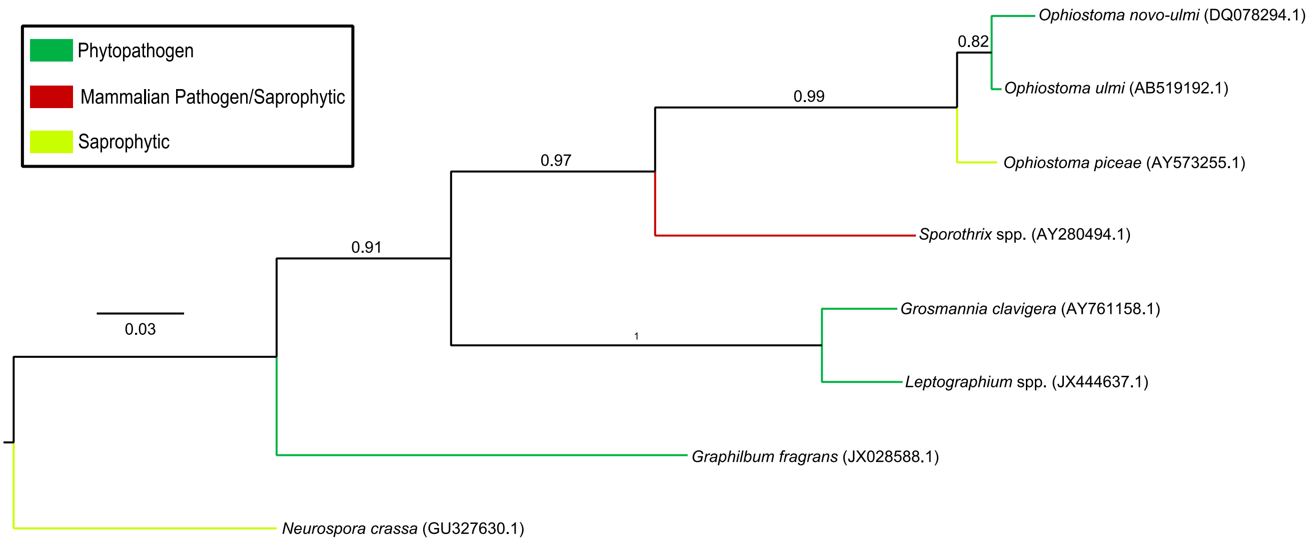

Figure 1: Maximum-likelihood tree based on the sequences of the 18S ribosomal RNA gene (partial), internal transcribed spacer 1, 5.8S ribosomal RNA gene, internal transcribed spacer 2 and 28S ribosomal RNA gene (partial). This tree was built to clarify the relationships among the Ophiostomataceae species used in comparative genomic analysis. Since the Ophiostomataceae family is closely related to the Sordariaceae family, a *Neurospora crassa* entry was added and the resulting tree was rooted at this species (outgroup). The orthologous sequences were classified according to fungal lifestyle trait, represented by different colors.
