# Supplementary material for: Genome-Wide Analysis of Secondary Metabolite Gene Clusters in Ophiostoma ulmi and Ophiostoma novo-ulmi Reveals a Fujikurin-Like Gene Cluster with a Putative Role in Infection
Source: Front Microbiol. 2017 Jun 13;8:1063. doi: 10.3389/fmicb.2017.01063 (PMC5468452; doi:10.3389/fmicb.2017.01063)
Supplement: Supplementary file 10 [file Image_4.PDF]

Supplementary Figure 4

# Genome-wide analysis of secondary metabolite gene clusters in *Ophiostoma ulmi* and *Ophiostoma novo-ulmi* reveals a fujikurin-like gene cluster with a putative role in infection

**Nicolau Sbaraini [1, 2], Fábio Carrer Andreis [1, 2], Claudia Elizabeth Thompson [1, 2, 3], Rafael Lucas Muniz Guedes [1, 3], Ângela Junges [2], Thais Campos [2], Charley Christian Staats[1, 2], Marilene Henning Vainstein [1, 2], Ana Tereza Ribeiro de Vasconcelos [1, 3], Augusto Schrank [1, 2,*].**

*** Correspondence:**
Augusto Schrank
aschrank@cbiot.ufrgs.br

Comparative genomic analysis and synteny for the type III PKS and siderophore compound BGCs and locus vicinity analysis for OpPKS8 BGC.

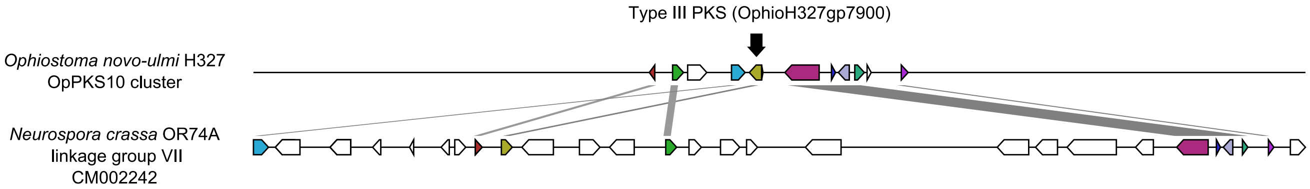


**Figure 1: Comparative genomic analysis and synteny for the type III PKS BGC (OpPKS10).** The backbone gene (OphioH327gp7900) is highlighted.

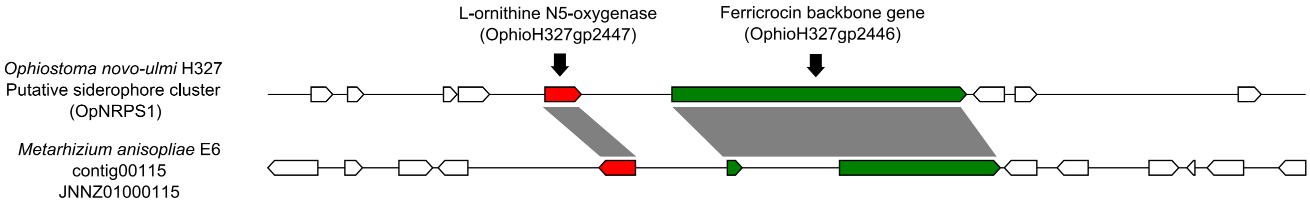


**Figure 2: Comparative genomic analysis and synteny for the putative ferricrocin BGC (OpNRPS1).** The ferricrocin backbone gene (OphioH327gp2446) and the L-ornithine N5-oxygenase gene (OphioH327gp2447) are highlighted.

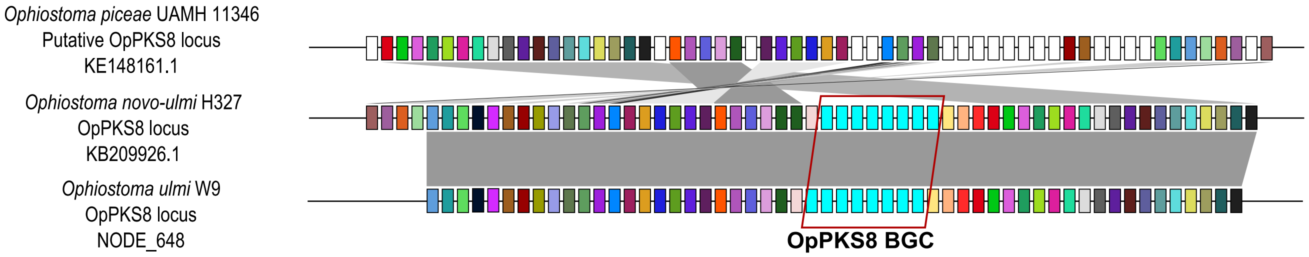


**Figure 3: Locus vicinity analysis for OpPKS8 BGC.** To further confirm the absence of OpPKS8 in *O. piceae* UAMH 11346, the vicinities of OpPKS8 locus were explored. The results confirmed the

absence of OpPKS8 gene cluster in *O. piceae* UAMH 11346, once, several genes in the vicinity have been conserved despite OpPKS8 gene cluster absence. The OpPKS8 BGC is highlighted. White blocks in *O. piceae* UAMH 11346 locus represent genes not conserved in *O. novo-ulmi* H327 and *O. ulmi* W9 loci, not necessarily implying in orthologs absence in the genomes (These genes can be located in other loci).
