# Supplementary material for: Genome-Wide Analysis of Secondary Metabolite Gene Clusters in Ophiostoma ulmi and Ophiostoma novo-ulmi Reveals a Fujikurin-Like Gene Cluster with a Putative Role in Infection
Source: Front Microbiol. 2017 Jun 13;8:1063. doi: 10.3389/fmicb.2017.01063 (PMC5468452; doi:10.3389/fmicb.2017.01063)
Supplement: Supplementary file 11 [file Image_5.PDF]

Supplementary Figure 5

Genome-wide analysis of secondary metabolite gene clusters in *Ophiostoma ulmi* and *Ophiostoma novo-ulmi* reveals a fujikurin-like gene cluster with a putative role in infection

Nicolau Sbaraini [1, 2], Fábio Carrer Andreis [1, 2], Claudia Elizabeth Thompson [1, 2, 3], Rafael Lucas Muniz Guedes [1, 3], Ângela Junges [2], Thais Campos [2], Charley Christian Staats[1, 2], Marilene Henning Vainstein [1, 2], Ana Tereza Ribeiro de Vasconcelos [1, 3], Augusto Schrank [1, 2,*].

*** Correspondence:**
Augusto Schrank
aschrank@cbiot.ufrgs.br

Schematic Ascomycota tree of life

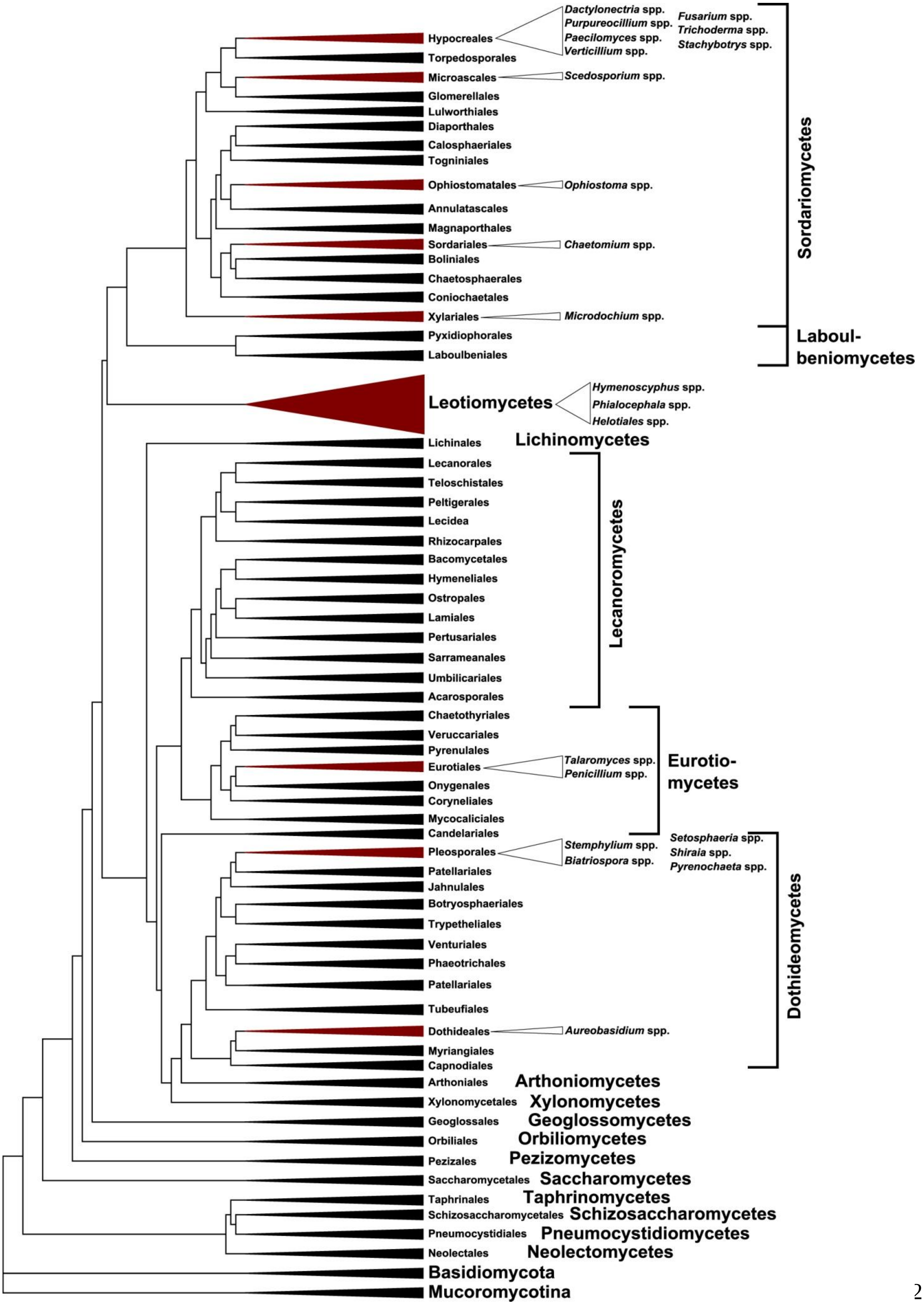
Hypocreales
Dactylonectria spp.
Purpureocillium spp.
Paecilomyces spp.
Verticillium spp.
Fusarium spp.
Trichoderma spp.
Stachybotrys spp.
Torpedosporales
Microascales
Scedosporium spp.
Glomerellales
Lulworthiales
Diaporthales
Calosphaeriales
Togniniales
Ophiostomatales
Ophiostoma spp.
Annulatascales
Magnaporthales
Sordariales
Chaetomium spp.
Boliniales
Chaetosphaerales
Coniochaetales
Xylariales
Microdochium spp.
Sordariomycetes
Pyxidiophorales
Laboulbeniales
Laboul-
beniomycetes
Leotiomycetes
Hymenoscyphus spp.
Phialocephala spp.
Helotiales spp.
Lichinales
Lichinomycetes
Lecanorales
Teloschistales
Peltigerales
Lecidea
Rhizocarpales
Bacomycetales
Hymeneliales
Ostropales
Lamiales
Pertusariales
Sarrameanales
Umbilicariales
Acarosporales
Lecanoromycetes
Chaetothyriales
Veruccariales
Pyrenulales
Eurotiales
Talaromyces spp.
Penicillium spp.
Onygenales
Coryneliales
Mycocaliciales
Eurotio-
mycetes
Candelariales
Pleosporales
Stemphylium spp.
Biatriospora spp.
Setosphaeria spp.
Shiraia spp.
Pyrenochaeta spp.
Patellariales
Jahnulales
Botryosphaeriales
Trypetheliales
Venturiales
Phaeotrichales
Patellariales
Tubeufiales
Dothideales
Aureobasidium spp.
Myriangiales
Capnodiales
Dothideomycetes
Arthoniales
Arthoniomycetes
Xylonomycetales
Xylonomycetes
Geoglossales
Geoglossomycetes
Orbiliales
Orbiliomycetes
Pezizales
Pezizomycetes
Saccharomycetales
Saccharomycetes
Taphrinales
Taphrinomycetes
Schizosaccharomycetales
Schizosaccharomycetes
Pneumocystidiales
Pneumocystidiomycetes
Neolectales
Neolectomycetes
Basidiomycota
Mucoromycotina

Figure 1. Schematic Ascomycota tree of life. The main fungal orders and classes are indicated and the species presented in Figure 3 are displayed (orders of these fungal species were colored). It is important to note that the orders sheltered in the Leotiomycetes class were collapsed. This tree was based in the work of (Schoch *et al.*, 2009).
